# Occurrence and Nature of Off-Target Modifications by CRISPR-Cas Genome Editing in Plants

**DOI:** 10.1021/acsagscitech.1c00270

**Published:** 2022-03-03

**Authors:** Mark H. J. Sturme, Jan Pieter van der Berg, Lianne M. S. Bouwman, Adinda De Schrijver, Ruud A. de Maagd, Gijs A. Kleter, Evy Battaglia-de Wilde

**Affiliations:** † Wageningen Food Safety Research, Wageningen University and Research, P.O. Box 230, 6700 AE Wageningen, The Netherlands; ‡ Sciensano, Rue Juliette Wytsmanstraat 14, 1050 Brussels, Belgium; § Wageningen Plant Research, Wageningen University and Research, P.O. Box 16, 6700 AA Wageningen, The Netherlands

**Keywords:** CRISPR-Cas, genome editing, plants, off-target modifications

## Abstract

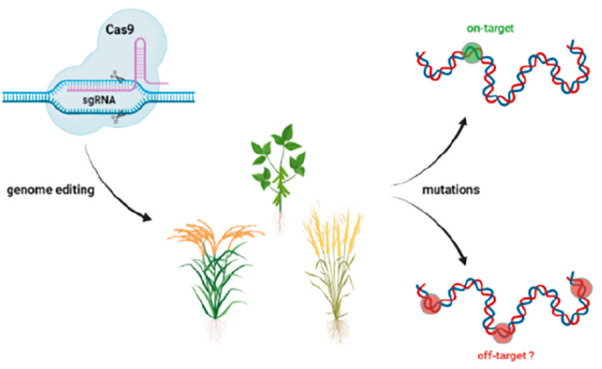

CRISPR-Cas-based genome editing allows for precise and targeted genetic modification of plants. Nevertheless, unintended off-target edits can arise that might confer risks when present in gene-edited food crops. Through an extensive literature review we gathered information on CRISPR-Cas off-target edits in plants. Most observed off-target changes were small insertions or deletions (1–22 bp) or nucleotide substitutions, and large deletions (>100 bp) were rare. One study detected the insertion of vector-derived DNA sequences, which is important considering the risk assessment of gene-edited plants. Off-target sites had few mismatches (1–3 nt) with the target sequence and were mainly located in protein-coding regions, often in target gene homologues. Off-targets edits were predominantly detected via biased analysis of predicted off-target sites instead of unbiased genome-wide analysis. CRISPR-Cas-edited plants showed lower off-target mutation frequencies than conventionally bred plants. This Review can aid discussions on the relevance of evaluating off-target modifications for risk assessment of CRISPR-Cas-edited plants.

## 1 Introduction

New genomic techniques, such as CRISPR-Cas-mediated (clustered regularly interspaced short palindromic repeat – CRISPR-associated protein) genome editing (also called gene editing), are molecular biology methods that can be used to introduce modifications at targeted genomic locations. These novel techniques have been developed over the last two decades and are increasingly being employed in the field of crop biotechnology, where they are also called new breeding technologies (NBTs). While NBTs comprise a range of techniques relying on site-directed nucleases (SDNs),^1,2^ in this study the focus is on targeted genome editing in plants by means of CRISPR-Cas tools. To date, the genomes of various crops, such as rice, tomato, maize, wheat, soybean, barley, potato, sorghum, apple, grapefruit, and orange, have been edited using CRISPR-Cas-based genome editing.^3^

The CRISPR-Cas technology was derived from the naturally occurring adaptive immune systems found in bacteria and archaea, where they function in defense against, as well as recognition and destruction of invading DNA (plasmids, bacteriophages).^4^ These systems are adaptive because they use an array of short sequences from earlier invading DNAs (the CRISPR array) to produce small RNAs (the guide RNAs or gRNAs) that function as a memory of past infections for a faster and more effective response. The gRNA, by being complementary to target DNA, allows for the specific recognition and cleavage (and neutralization) of the target DNA by a nuclease, which consists of one or more Cas proteins. Many CRISPR-Cas systems exist in nature, of which only a few are exploited in biotechnology, but this number is growing.^5^

Several of these systems consist of multiple protein components. Especially relevant and used for biotechnology applications are the type II (Cas9), and to a lesser extent type V (Cpf1/Cas12a) nucleases, because these combine gRNA binding, target recognition, and cleavage in a single protein.

**Figure 1:**
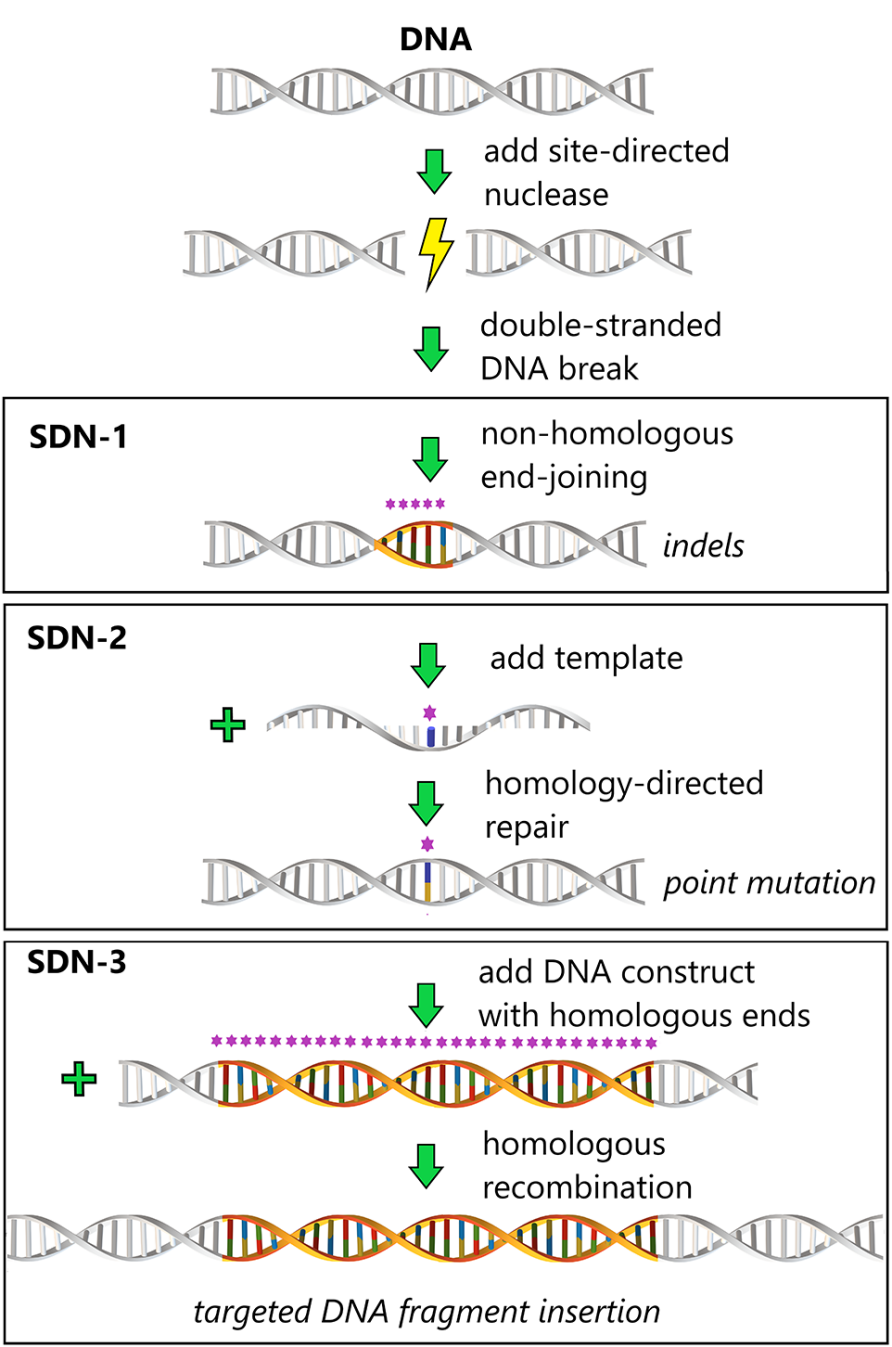
Schematic overview of the types of SDN modifications. The asterisks (*) signify nucleotides (in color) that are not identical to the native host sequence (in gray) around the double-stranded break introduced by the SDN. Such nonidentical nucleotides are introduced either through substitution or through insertion of nucleotides during the process of DNA break repair. SDN-1 applications can generate 1 base pair (bp) up to a small number of base insertions/deletions (indels) without providing a donor DNA template, through nonhomologous end-joining (NHEJ). Occasionally larger deletions may occur as a result of alternative repair mechanisms such as microhomology-mediated end-joining (MMEJ). SDN-2 applications can generate precise and small genetic modifications at the target site, ranging from point mutations to small indels, by means of a donor DNA template for homology-directed repair (HDR). SDN-3 applications can insert entire DNA cassettes into a target site, by providing a large donor DNA template of the desired gene, which leads to insertion by HDR or NHEJ and a transgenic plant if the donor originates from an unrelated species.

Besides CRISPR-Cas, other site-directed nucleases, such as zinc-finger nucleases (ZFNs), transcription activator-like effector nucleases (TALENs) and meganucleases, have been used to achieve SDN-1, SDN-2, and SDN-3 mutations (see Figure 1), but in recent years these are increasingly replaced by CRISPR-Cas.^6−8^ More recently, new CRISPR-based genome editing tools such as base editors have been developed and used to modify crops.^9^ Base editors are used to introduce specific nucleotide substitutions in a targeted sequence without inducing double strand breaks (DSBs) in the target locus or using template DNA. There are currently three types of DNA base editors: the cytosine base editor (CBE), the adenine base editors (ABE), and the dual base editors that enable single-base substitutions. CBEs and ABEs are composed of a fusion between a catalytically impaired Cas nuclease domain (Cas9 variants, a dead Cas9 or Cas9 nickase) and a base-modification enzyme, either the domain of a cytosine deaminase or an adenine deaminase.^9^ CBEs and ABEs generate C·G-to-T·A or A·T-to-G·C conversions, respectively. Dual base editors are catalytically impaired Cas9 variants to which both the cytosine and adenine deaminase are fused, which allows for simultaneous cytosine and adenine base editing.^1,10^

Modification of genomes using CRISPR-Cas and derived base-editor tools only requires the guide RNA sequence to be adapted for each DNA target site. Therefore, it is a simple and versatile tool for genome editing. Unlike other mutagenesis and genetic engineering methods, such as chemical and irradiation mutagenesis or transgenesis, CRISPR-Cas genome editing allows for the introduction of precise and predictable small modifications, especially with Cas9 variants with higher on-target specificity^11^ into an elite variety genetic background.^12^ However, off-target modifications, which are usually defined as changes to the DNA or RNA, in regions other than the target site, are known to occur as a consequence of gene editing despite the specificity of the Cas9 enzyme and other CRISPR-associated endonucleases.

In biomedical research that focuses on the potential use of CRISPR-Cas as a therapeutic tool for human diseases, considerable effort has been put into improving target specificity of CRISPR-Cas-mediated genome editing in order to greatly reduce the chance of off-target activity.^11^ This has been done to a lesser extent in the field of plants. The most frequently used Cas-nuclease in plant breeding is Cas9, while Cas12a (Cpf1) has been used less often.^7^ The scientific literature concerning off-target modifications caused by CRISPR genome editing in plants has been analyzed and discussed in several peer-reviewed reviews and research studies over the last years.^3,7,13−19^ Two of these studies are systematic literature reviews by Modrzejewski and colleagues: one in-depth literature study on the range of applications of genome-editing in plants and the occurrence of off-target modifications^7^ and one analysis of factors affecting the occurrence of off-target modifications caused by CRISPR-Cas genome editing.^3^ As highlighted in these two studies, there is a lack of unbiased genome-wide off-target analysis and therefore still several knowledge gaps concerning genome editing in plant breeding and the occurrence of off-target modifications exist, such as the role of the number and position of mismatches with the guide RNA, of the G+C-content of the targeting sequence, and of altered nuclease variants and their delivery mode. Addressing these aspects further by in-depth studies would shed a better light on the occurrence and the detection of off-target modifications.

The aim of this Review is to provide a detailed overview of the published observed off-target DNA modifications caused by CRISPR-associated endonucleases in crop plants by means of a literature search and analysis. In this literature search, off-target DNA modifications induced by Cas9, and other Cas enzymes such as Cas12a/Cpf1, Cas variants or the CRISPR-guided base editors are considered. The off-target modifications in different plant species are listed and described in detail, including the following parameters (if mentioned): the CRISPR-Cas tool used, the detection method used to identify potential off-target changes, the number of mismatches found between the gRNA and off-target site(s), the type of off-target modification found (insertion or deletion and their size, nucleotide substitution), and the location of the off-target modification (coding or noncoding region, and if specified, the gene name and its accession number).

## 2 Literature Search

The systematic literature search followed a sequential approach comprising of two stages with different (sub)steps. These steps follow the methodology described elsewhere for systematic reviews, albeit that the current search was more flexible, geared toward the comprehensive inclusion of all relevant aspects and information. Details of the search strategy and data collection and analysis procedures can be found in Supplementary File 1. The first stage consisted of defining the review goals, followed by search string formulation. In the second stage, data were collected through bibliographic searches and collation of retrieved records from databases. Relevant references were selected in two substeps: (1) an initial title-abstract screening based on inclusion criteria and (2) retrieval of full references followed by an in-depth screening based on inclusion criteria. Irrelevant references were disposed during these two substeps. Relevant references were selected for full-text data mining (i.e., critical appraisal and evidence mapping) and in a final step data extracted and summarized.

### 2.1 Research Question and String Formulation

The questions that the literature survey sought to answer were the following: what is reported in literature about (1) the nature and frequency of off-target mutations caused by genome editing tools in crops? and (2) the potential food and feed safety hazards or risks linked to side effects of genome editing used for creating small mutations in crops?

These questions engendered different concepts, as follows: (1) Intervention: side-effects of genome editing of a host crop: the terms used for searching through the selected bibliographies should cover (a) the various types and synonyms for genome editing methods (e.g., CRISPR-Cas9, TALEN, ZFN) that can be applied, as well as (b) the various food and feed crops that may be modified (trivial and generic species names) and (c) side effects of the gene edit (for example, off-target modifications), (2) Comparator: nonedited, conventional crops currently used as food or feed with a history of safe use, (3) Population: Consumers (human and animal) of the edited crops and (4) Outcomes: health hazards and risks for consumers (for example, negative health effects, adverse reactions, toxicity, allergenicity).

### 2.2 Collection of Data and Full-Text Screening of the Relevant Literature

Data were collected from both scientific literature and from “grey” sources. Data from peer-reviewed scientific literature was collected from the databases Web of Science, Scopus, and Centre for Agriculture and Bioscience International (CAB) using the search strings described in Supplementary File 1. The settings were adjusted to include only the 5 most recent years of publication given that the developments are progressing fast and that developments in CRISPR-Cas9 technology started to evolve after the year 2012. Abstracts and retrieved records from these databases were screened for their relevance, based on inclusion criteria, before retrieval of full references and in-depth screening. The snowballing approach was then used to search for further relevant literature from the references in literature reviews collected in our initial search. For critical appraisal of the relevant CRISPR plant studies, we used the following parameters: CRISPR tool used, method of delivery of CRISPR components, the number of allowed mismatches to search for potential off-target sites, the number and type of off-target modification(s) found, including the size of the indel(s) and the location of the off-target modification. The extracted data were used for a narrative description of the outcomes. The “grey” sources included opinions and assessments from international risk assessment bodies specialized in the food/feed safety assessment of new and gene-edited/genetically modified crops. These sources were scanned for information on potential off-target modifications from genome editing identified in gene-edited plants evaluated by these bodies. Moreover, it was checked if and which possible effects for health and safety of food and feeds produced from these crops had been assessed.

## 3 DETAILS OF OFF-TARGET MODIFICATIONS IN CRISPR-CAS GENE-EDITED PLANTS

### 3.1 Occurrence and Nature of Off-Target Modifications

A systematic literature search gathered a total of 107 publications that were analyzed in-depth and evaluated for the occurrence, frequency, and type of off-target modifications caused by CRISPR endonucleases in different plant species (Supplementary Table 1). The review of the selected literature shows that screening for off-target gene edits is often performed in the fundamental research phase to establish the specificity of the guide RNAs used for a particular goal, rather than the specificity of the method or nuclease in general. To analyze the strategies for the identification of potential off-target genome edits, we made a distinction between the so-called “unbiased” and “biased” methods. Unbiased methods are defined as methods based on the genome-wide screening for small DNA modifications or DSBs, which can be reliably connected to the nuclease activity. Biased methods involve targeted analysis of possible DNA mutations at selected genome sites that were predicted to be potential off-target sites by *in silico* methods. It was observed that whole genome sequencing (WGS) is rarely used to analyze off-target sites for true off-target mutations (7.5%; 8/107). The majority of studies (72%; 77/107) made use of specific bioinformatic tools such as CRISPR-P (http://crispr.hzau.edu.cn/CRISPR2/) and Cas-OFFinder (http://www.rgenome.net/cas-offinder/) for the design of gRNAs and prediction of potential off-target sites.^20,21^ Alternative methods for the prediction of potential off-target sites are based on, for instance, the use of basic local alignment search tools (BLAST) or selection and analysis of close homologues of the on-target site containing gene. In 92% (98/107) of the selected studies, predicted off-target sites were amplified by PCR, and the amplicons were further analyzed by means of sequencing and alignment with reference sequences, or by enzymatic digestion analysis, for the presence of DNA modifications.

Studies that performed unbiased detection of off-target editing often went a step further and also compared the outcome of the WGS to predicted off-target sites to analyze whether identified mutations were spontaneous mutations or resulting from genome editing.^22−25^

The number of potential off-target sites predicted by specific bioinformatic tools depends on the settings used for the prediction. In the selected studies, certain features of the gRNA and complementary DNA site were specified, such as the maximum number of mismatches allowed between the off-target site and complementary sequence of the gRNA and/or the presence and maximum size of DNA and RNA bulges. Of the 94 studies that performed a bioinformatics prediction of off-target sites, only 32 described the maximum amount of nucleotide mismatches allowed between the complementary gRNA sequence and potential off-target sites, with the majority (28/32) of studies setting a maximum mismatch of 5 nucleotides in their analysis of potential off-target sites. No off-target modifications caused by CRISPR endonucleases were reported in 63% of the studies (67/107). In 11% (12/107) of the studies, the outcome of the off-target analysis was not reported. In 26% (28/107) of the selected literature, modifications caused by CRISPR endonucleases were reported (Table 1). Genome editing in these studies was predominantly done with wild-type or codon-optimized *Streptococcus pyogenes* Cas9 (SpCas9) and in two cases with Cas12a (Cpf1) from Lachnospiraceae bacterium (LbCas12a) or *Francisella novicida* (FnCpf1). Base editing was done using the Cas9 nickase variant (nCas9) and epigenome editing using a catalytically inactive Cas9 variant (dCas9). The main off-target modifications for Cas9 and Cas12a reported in 10 studies consisted of small indels (1–22 nucleotides in size) and nucleotide substitutions, while 13 studies mentioned indels without further details. In addition, the insertion of large vector-derived sequences in the target site was observed in one study,^26^ and nonspecific on-target edits (On-T-ns) were observed in two studies, namely, 1 bp indels, nucleotide substitutions, and larger deletions.^27,28^ Off-target modifications for base editors (4 studies) consisted of base edits without other types of modifications reported. One study described the off-target effects for a dCas9 methyltransferase system, which led to unintended genome-wide effects such as CHH hypermethylation (where H = A, T, or C) and chloroplast DNA methylation.^29^ Most studies reported off-target indels and nucleotide substitutions in (predicted) off-target protein-coding sequences in the genome. Two studies that used an unbiased method, also reported off-target indels and nucleotide substitutions in noncoding regions of the genome.^22,30^ In 18 out of 28 studies, an indel was observed to occur in a coding region, which in 10 cases was in genes homologous to the gRNA targeting gene. These off-target sites exhibited 1 to 3 mismatches with respect to the gRNA. For the remaining 10 studies, the specific location of the off-target modification in the plant genome was not specified.

**Table 1:** Details of the Reported off-Target and Unwanted on-Target Changes by CRISPR-Cas Genome Editing in Plant Species for the 28 Peer-Reviewed Studies Performing an Off-Target Analysis

plant species	Cas variant	off-target detection method: biased (B) unbiased (U)	description of method (s)	target gene	off-target or unwanted on-target changes^a^	number of mismatches off-target with gRNA	indel size (bp) and frequency or change at off-target/on-target location	location (coding/noncoding)	off-target gene	reference
Apple (*Malus domestica*), pear (*Pyrus communis*)	Cas9	B	*In silico* prediction followed by PCR amplification and sequencing	TFL1	“Off-T”^b^	0^b^	Insertions: +1 (1x), +7 (1x)	Coding	PEBPMD12	(44)
							Deletions: –1 (8x), −2 (2x), –4 (1x), −6 (1x)			
*Arabidopsis thaliana*	A dCas9-SunTag system; dCas9 with the catalytic domain of the *Nicotiana tabacum* DRM methyltransferase (NtDRMcd)	U	Whole genome bisulfite sequencing (WGBS) to screen DNA methylation	FWA	Off-T: genome-wide epigenetic off-target effects were observed such as CHH hypermethylation (where H = A, T, or C) and chloroplast DNA methylation	Not specified	DNA methylation	Not specified	Not specified	(29)
*Arabidopsis thaliana*	Cas9	B	Digenome-seq and targeted amplification deep sequencing of potential off-target sites	TRY	Off-T	2	Insertions: +1 (88.4–90.7%)	Coding	Not specified	(45)
							Deletions: –1 (2.2–3.1%)			
Barley (*Hordeum vulgare*)	Cas9	B	*In silico* prediction followed by PCR/Sanger sequencing	HvPM19-1	Off-T	1	Indels: size not specified	Coding	HvPM19–3 (target homologue)	(32)
*Brassica oleracea*				BolC.GA4.a	Off-T	2	Indels: size not specified	Coding	BolC.GA4.b (target homologue)	
Cassava (*Manihot esculenta*)	Cas9	B	PCR amplification of 504 bp of the target sequence followed by Sanger sequencing	MePDS	On-T and On-T-ns	1	Mainly 1 bp insertions (+1) and deletions (−1) on-target. Nucleotide substitutions also indicated on-target, but outside of target site. Deletions of 16 bp and 101 bp also observed	Coding	MePDS (on-target CDS)	(27)
	Cas9	B	*In silico* prediction followed by PCR amplification and sequencing	nCBP-2	Off-T	2 or 3	Deletions: −1, −3, and −11 bp	Coding	Not specified	(46)
Cotton (*Gossypium hirsutum*)	Cas9	U	Whole genome sequencing (WGS), assessment of off-target mutations at predicted potential off-target sites	MYB44 and ARC	Off-T	3 (for MY44) and 2 (for ARC)	Indels of 1–4 bp at Crd1, 1-bp at MYB77.	Coding and noncoding	Promoter dicarboxylate diiron gene (Crd1. First exon of MYB77 (MYB44 target homologue)	(22)
							1-bp deletions with the ARC gRNA.			
	nCas9 cytidine base editing system	U	Whole genome sequencing as well as targeted deep sequencing of potential off-target sites	GhCLA and GhPEBP	Off-T	1 to 5	Base edit: less than 0.1% single nucleotide substitutions	Coding	Not specified	(47)
Maize (*Zea mays*)	Cas9	B	A three-step approach: (1) *in silico* prediction, (2) combination of in silico predictions with CLEAVE-seq (a genome-wide biochemical assay) data, (3) off-target site monitoring using Molecular Inversion Probe (MIP) analysis	MS26, MS45, Lig1	Off-T	1 or 2	Not specified	Not specified	Not specified	(25)
	Cas9	B	*In silico* prediction followed by PCR and deep sequencing	MS45	Off-T	3 (2 in gRNA, 1 in PAM)	Not specified	Not specified	Not specified	(38)
Plantain (*Musa sp.)*	Cas9	B	*In silico* prediction followed by PCR amplification and sequencing	BSOLV/eBSOLV	Off-T	2	Point mutation (not specified)	Coding	Ma01_t10610.1	(48)
Rice (*Oryza sativa*)	Cas9	B	*In silico* prediction and restriction enzyme digestion suppressed PCR (RE-PCR) combined with Sanger sequencing	PS3	Off-T	3	Deletion, size not specified	Coding	Not specified	(49)
	Cas9 and LnCas12a	U	WGS complemented by an *in-silico* prediction, PCR amplification and Sanger sequencing of the selected sites	Os02circ25329	Off-T	1 to 3	Deletions: 1–22 bp (including in PAM)	Not specified	Not specified	(23)
	Cas9	B	*In silico* prediction followed by PCR amplification and sequencing	OsYSA sgRNA2	Off-T	1	Not specified	Not specified	Not specified (location Chr11:1535478–1535497)	(50)
	Cas9	B	*In silico* prediction followed by PCR amplification and sequencing	8 genes	Off-T	2 and 6	Not specified	Not specified	Not specified	(51)
	nCas9 adenine-base editing system	B	*In silico* prediction followed by PCR amplification and sequencing	OsSPL14	Off-T	1	Base edit	Coding	OsSPL17 (target homologue)	(34)
	Cas9	U	WGS, assessment of small indels and SNVs most likely to be true positives	IAA and ARF	Off-T	Not specified	Indels and nucleotide substitutions	Coding and noncoding	Os02g0618200 and Os10g0147400	(30)
	Cas9-NG	B	*In silico* prediction followed by PCR amplification and sequencing	OsMPK10- and OsMPK11	Off-T	1 (in PAM)	Insertions and deletion of 1-bp	Coding	OsMPK9 (target homologue)	(35)
	nCas9 base editing system	B	*In silico* prediction followed by PCR amplification and sequencing	ALS	Off-T	1 to 3	Base edit	Not specified	Not specified	(52)
	FnCpf1	B	Homologous gene selection and sequencing	OsNCED1 and OsAO1	Off-T	1	Deletion	Coding	OsNCED2 and OsAAO4 (target homologues)	(33)
	Cas9	B	PCR followed by enzymatic digestion analysis	OsMPK2 and OsPDS	On-T-ns	1 or 3	Large deletion in on-target gene, but outside of target site	Coding	PDS_NI-1, MPK2_NI-1 and MPK2_NI-2	(28)
Soybean (*Glycine max*)	Cas9	B	*In silico* prediction followed by PCR amplification Sanger sequencing	Glyma11g07220 (DDM1) and miR1514	Off-T	2	Indels: deletions most common, SNPs less common	Not specified	Not specified	(53)
Soybean (*Glycine max*)	Cas9	B	*In silico* prediction followed by PCR amplification Sanger sequencing	Glyma06g14180 and Glyma12g37050	Off-T	1	Insertions: +1 (18x), +2 (3x), +5 (1x)	Coding	Glyma04g40610 and Glyma09g00490	(54)
							Deletions: −1 (1x), −3 (2x), −4 (1x)			
							Some nucleotide substitutions as well			
Tomato (*Solanum lycopersicum*) and potato (*Solanum tuberosum*)	nCas9 cytidine base editing system	B	Homologous gene selection and sequencing	SlALS1	Off-T	1	Base-edit	Coding	SlALS2 (target homologue)	(55)
Wanjincheng orange (*Citrus sinensis Osbeck*)	Cas9	B	*In silico* prediction followed by PCR amplification and sequencing	CsLOB1 promoter	Off-T	2 to 4	Base edit: 1-bp substitutions	Not specified	Not specified	(56)
	Cas9	U	WGS in conjunction with *in silico* prediction	2 CsWRKY22 alleles	Off-T	<5	Indels and nucleotide substitutions	Not specified	chr8:-14917433, chr2:-8217719, chr9:-4784585, chr9:-14360722	(24)
Wheat (*Triticum aestivu*m)	Cas9	B	*In silico* prediction followed by PCR and enzymatic digestion analysis	TaGW2 homologues	Off-T	1	Insertions: some +1	Coding	TaGW2 target homologues	(36)
							Deletions: mainly −1 to −9			
	Cas9	B	Deep sequencing of homeoalleles of the target gene	7AS-EPSPS	Off-T	1	Off-T: Majority deletions (1–10 bp), one 1-bp insertions. *Many large insertions (>20bp) of vector-derived sequences*	Coding	7DS-EPSPS (target homologue)	(26)

a Off-target (Off-T) = modification outside of target gene. On-target nonspecific (On-T-ns) = modification inside of target gene, but outside of target site.

b Charrier et al.^44^ defined a modification in a nontarget gene at a site with 0 mismatches as off-target. In our opinion a gene edit at a site with 0 mismatches by definition should be called on-target.

### 3.2 Frequency of Off-Target Modifications

Besides the occurrence and nature of off-target edits resulting from genome editing, it is also important to know the frequency at which these edits are introduced. From our literature review, three CRISPR-Cas plant studies were identified in which a genome-wide identification of off-target mutations was performed and subsequently compared to background mutations.^22,23,25^ These three studies observed that off-target mutations caused by Cas9 occur at a much lower level than background mutations such as genetic changes due to soma-clonal variation. Tang et al.^23^ used WGS to compare rice plants edited with Cas9 or Cpf1 to control plants. They observed that the majority of mutations found, approximately 102 to 148 single nucleotide polymorphisms (SNPs) and approximately 32 to 83 indels per plant, were the result of soma-clonal variation. Furthermore, they found no Cas9- or Cpf1-induced off-target mutations in 47 out of 49 analyzed rice plants. In the remaining two plants, off-target mutations (indels in 12 off-target sites) were observed for only one gRNA out of 12 gRNAs. This particular gRNA exhibited significant similarity to the off-target sites, as most of these 12 sites showed only one nucleotide mismatch in the protospacer sequence. They predicted these off-target sites beforehand using online bioinformatic tools CRISPOR (http://crispor.tefor.net/) and Cas-OFFinder (http://www.rgenome.net/cas-offinder/). Li et al.^22^ used WGS to study the Cas9-induced off-target edits in cotton plants compared to somaclonal variation and inherent genetic variation. The WGS data revealed only four Cas9-induced off-target indels, with mutations present in 2 out of 182 predicted off-target sites for MYB44 sgRNA2 and 2 out of 341 for ARC sgRNA1, while higher numbers of spontaneous mutations were observed: an average of 466 SNPs and 77 indels per plant. Young et al.^25^ did not use WGS but described a targeted three step approach to identify Cas9 induced off-target mutations. One of the steps consisted of their novel biochemical method CLEAVE-Seq, for the identification of candidate off-target sites. Off-target mutations were only observed in the Cas9-edited maize plants in which a gRNA was used that was intentionally selected for its potential of inducing off-target edits. They also analyzed the natural variation in their maize control plants at the candidate off-target sites and showed SNPs were generated at these sites as a result of either inherent or spontaneous variation. They concluded that, with appropriately designed guide RNAs, off-target mutations are likely to be negligible in the background of existing natural variation.

## 4 Discussion

Off-target modifications are seen as an undesired byproduct of CRISPR-Cas genome editing in plants. Although the occurrence of these side effects has been mentioned in many studies, full details on for instance the frequency, nature and location of the modifications are often not analyzed or described in research papers. In addition, factors that contribute to the occurrence of off-target modifications have not been described in detail, and information on potential phenotypical effects is lacking. In this study, we therefore set out to perform a systematic literature search to collect studies on genome editing in agricultural plants that describe the details of off-target modifications.

Our in-depth analysis confirms that although many studies mention the occurrence of off-target modifications, only a limited number provide further (molecular) details. The 28 studies describing off-target modifications covered a variety of 12 crop species as well as *A. thaliana*, but many studies (10 out of 28) focused on genome editing in the important cereal rice. The available literature for these species indicates that off-target modifications in general consist of small insertions or deletions (1–22 bp) or single nucleotide substitutions at the DSB site and that the observed number of mismatches of the off-target sequence with the gRNA in most cases was 1–3 bp. The detection of single nucleotide substitutions might not be expected for the Cas9 nuclease without base editing capacity and could be attributed to sequencing errors. However, a few studies have described that single nucleotide substitutions can result from the activity of Cas9.^31^

Off-target modifications were more often located in protein-coding regions than noncoding regions. In many cases, these protein-coding regions were homologues of the target gene, as shown for e.g. barley,^32^*Brassica oleracea*,^32^ rice^33−35^ and wheat.^26,36^ This is an expected outcome, as plants may have many alleles/gene-homologues with the same function that often show little sequence diversity. Our findings are complementary to a recent study by Modrzejewski et al.,^3^ in which a systematic literature analysis was performed to assess the occurrence of off-target effects for CRISPR-Cas genome editing in plants. They demonstrated that an increased number of mismatches between the on-target sequence and the potential off-target sequence steeply decreases the likelihood that off-target effects occur. The observed rate of off-target effects decreased from 59% for one mismatch between the on-target and off-target sequences toward 0% when four or more mismatches were present. The position of the mismatches with the gRNA is also known to have an effect on the occurrence of off-target modifications, but this information could not be obtained from the references in our study. However, Modrzejewski et al.^3^ indicate that there is a tendency that off-target effects are reduced when the mismatches are located within the first eight nucleotides proximal to the protospacer adjacent motif (PAM). Statistical meta-analysis in their study further indicated that the position of the mismatches significantly affects the occurrence of off-target effects but less strong compared with the number of mismatches of the on-target and off-target sequences. The study also mentioned that there was no evidence that the G+C content of the genome significantly affects off-target editing.

Other factors that could play a role in the occurrence and frequency of off-target modifications are the type of side-directed nuclease used and the delivery mode of the CRISPR-Cas machinery. Data regarding the impact of these factors are still poorly understood as the majority of studies applied the widely used SpCas9 nuclease and not e.g. Cas12a (Cpf1). These two Cas variants have different properties for e.g. editing efficiency, sequence specificity and DNA cleavage outcome, and thereby both advantages and disadvantages for genome editing.^37^ Therefore, by testing both variants, one could select the Cas variant with the highest on-target and lowest off-target activity for a particular plant species. The CRISPR-Cas system was also often stably integrated in the genome, while there are indications that delivery of the CRISPR gRNA and Cas nuclease molecules directly as a ribonucleoprotein (RNP) complex and not on a DNA vector can reduce the frequency of off-target modifications, as for example was described by Svitashev et al.^38^

Insertion of vector-derived DNA sequences in off-target locations was observed in one of the studies.^26^ This is an important finding that can have regulatory consequences, as introduction of these foreign DNA sequences will qualify a new plant variety as a transgenic plant instead of a gene-edited plant. If only limited molecular analysis is done without screening for such vector DNA insertions for gene-edited plant varieties (when the CRISPR-Cas is introduced on a DNA vector), these insertions might go unnoticed. The risk of such vector DNA integrations at off- and on-target sites could be eliminated by delivering the gRNA and Cas nuclease directly as a RNP complex.

Many studies only performed a biased screening of the most likely predicted off-target sites in a limited number of plants, and therefore might detect a lower number of off-target modifications and lower mutation frequencies than are actually present in the genome. Therefore, when it comes to off-target analysis during development of gene-edited plants, unbiased off-target detection methods, like WGS, would be preferred, as they allow for the detection of off-target changes in genomic regions (both coding and noncoding) that were not identified by in silico off-target predictions.

Besides undesired off-target modifications and insertion of vector-derived DNA sequences, also unintended on-target edits in plants were observed in two studies^27,28^ (see Table 1). Although the respective studies described these unintended events as off-target effects, we defined them as on-target but nonspecific (On-T-ns), as the modification was observed outside of the target site but in the target gene. Some of these modifications cannot be explained by the current knowledge of CRISPR-Cas editing mechanisms and require further investigation. Other unintended on-target DNA changes that can occur during CRISPR-Cas genome editing are for example large chromosomal fragment deletions leading to loss of heterozygosity, as well as large insertions and inversions,^39^ which are aspects that have not been studied in plants. Further, for CRISPR-guided base editors also transcriptome-wide RNA edits have been reported in human cells, and such edits might be expected in plants as well.^40,41^ Moreover, it has become clear that even an on-target, intended modification may not result in the intended effect. For example, a clean knockout of gene function may not be achieved, as partial gene function can be maintained due to alternative splicing or an alternative translation start or even genetic compensation by upregulation of a related locus may result in the maintenance of a partial gene function.^42^ However, our literature analysis did not find information on these potential additional unintended effects.

Off-target modifications but also nonspecific on-target modifications by genome editing are considered undesirable, and the genetic and biochemical consequences of these unintended modifications are issues that should be taken into account when developing genome editing tools for plant breeding purposes, as they could lead to unexpected effects on plant traits. The systematic literature review did not identify any studies that provided information on the biochemical and phenotypical effects of such modifications.

**Figure 2:**
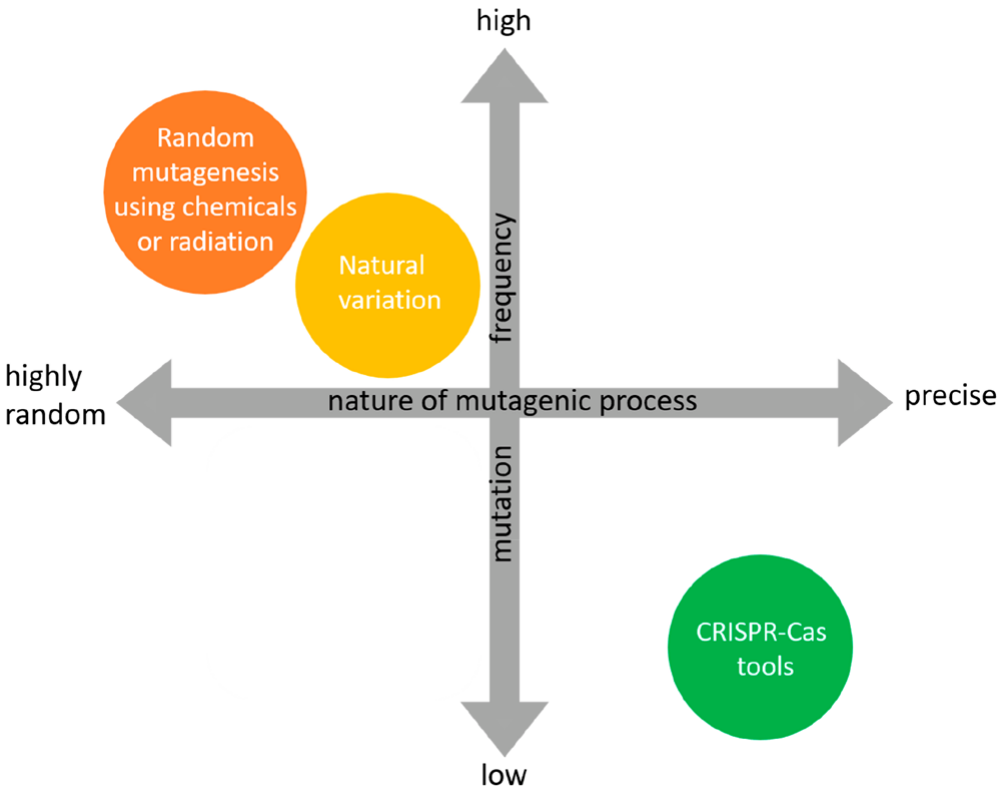
Schematic overview of the mutation frequencies of different plant breeding techniques, based on our literature study.

When discussing the potential risks of novel plant breeding techniques such as CRISPR-Cas genome editing, it is important to address the question whether the frequency of unintended DNA mutations arising from SDN-1 application of CRISPR-Cas technology differs from the frequency of such mutations arising from other plant breeding methods. Recently, Kok et al.^43^ presented a literature-based overview summarizing the mutation frequency and density of different plant breeding methods.^43^ Data on the mutation frequency for mutation breeding techniques, in which irradiation or chemical mutagens are used, is available but highly variable. Mutation frequency is dependent on many factors, such as the type of radiation or chemical mutagen and the dose used. For irradiation, reported mutation frequencies are typically in the range of 6.63 × 10^–3^ mutations per base pair (bp) to 3 × 10^–8^ mutations per bp. For chemical mutation breeding, a higher occurrence of mutations is frequently observed, with mutation densities ranging from 1 SNP per 0.05 kilobase (kb) to 1 SNP per 2500 kb. Chemical mutagenesis therefore induces at least 3-fold higher mutation frequencies than irradiation. Usually, the mutation density is calculated by dividing the total number of sequenced base pairs by the number of identified mutations through screening of selected genes in a population. For CRISPR-Cas and other new genome editing tools like ZFNs and TALENs, they observed, based on the literature search, that no or limited data are available on the mutation frequency and unintended mutations could be retrieved from literature.^43^ The available data on mutation frequency of different plant breeding techniques are summarized in a schematic figure (Figure 2). This figure is based on the results from our literature review and the mutation frequencies reported by Kok et al.^43^ All plant breeding techniques are placed relative to the level of spontaneous mutations occurring in plants due to soma-clonal variation or inherent genetic variation. It is evident from literature that mutagenic treatments result in a higher occurrence of mutations compared to the level of spontaneous occurring mutations. Thus, the mutation levels of these classical breeding methods are placed above the level of spontaneous occurring mutations in Figure 2. On the basis of the observations from the three plant studies described above in which unbiased methods were used, the occurrence level of CRISPR-induced off-target mutations was placed below the level of spontaneous mutations.

While we observed from literature that the chance of an off-target mutation occurring in the genome is low when using CRISPR-Cas genome editing, we have to keep in mind that this is based on a limited amount of available data. Only a small number of peer-reviewed studies are available in which the CRISPR-Cas-induced mutations are studied together with, and in two studies even distinguished from, naturally occurring or background mutations.^22,23,25^ Moreover, very few studies have used “unbiased” methods and a systematic approach to detect genome-wide off-target mutations.

In conclusion, in this study, we have performed an in-depth analysis of the available literature on off-target modifications by CRISPR-Cas genome editing in plants, and provided novel insights on the occurrence, frequency, and nature of off-target edits. Our analysis highlights the factors that might be improved to reduce off-target modifications and the information provided can serve as a basis for future discussions on the relevance of (and methods for) evaluating off-target modifications during risk assessment of CRISPR-Cas-edited plants.

## Supporting Information Available

Supplementary Text S1: Systematic literature research method (PDF)
Supplementary Table S2: Overview of the scientific literature that report the identification of potential off-target sites (PDF)

## ABBREVIATIONS USED

ABE: adenine base editor
CBE: cytosine base editor
Cas: CRISPR-associated protein
CRISPR: clustered regularly interspaced short palindromic repeat
DSB: double strand break
HDR: homology-directed repair
Indel: insertion or deletion
NBTs: new breeding technologies
PAM: protospacer adjacent motif
NHEJ: nonhomologous end-joining
SDNs: site-directed nucleases
SNPs: single nucleotide polymorphisms
TALENs: transcription activator-like effector nucleases
WGS: whole genome sequencing
ZFNs: zinc-finger nucleases
