# Supplementary material for: Occurrence and Nature of Off-Target Modifications by CRISPR-Cas Genome Editing in Plants
Source: ACS Agric Sci Technol. 2022 Mar 3;2(2):192–201. doi: 10.1021/acsagscitech.1c00270 (PMC9075866; doi:10.1021/acsagscitech.1c00270)
Supplement: Supplementary file 1 — as1c00270_si_001.pdf [file as1c00270_si_001.pdf]

Supporting Information: Systematic literature research method

Occurrence and nature of off-target modifications by CRISPR-Cas genome editing in plants

Mark H.J. Sturme[1*#], Jan Pieter van der Berg[1*], Lianne M.S. Bouwman[1], Adinda De Schrijver[2], Ruud A. de Maagd[3], Gijs A. Kleter[1], Evy Battaglia-de Wilde[1]

[1] Wageningen Food Safety Research, P.O. Box 230, 6700 AE Wageningen, The Netherlands

[2] Sciensano, Rue Juliette Wytsmanstraat 14, 1050 Brussels, Belgium

[3] Wageningen Plant Research, P.O. Box 16, 6700 AA Wageningen, The Netherlands

[*] Equal contribution

[#] Corresponding author: mark.sturme@wur.nl

Supplementary Text S1: Systematic literature research method

The literature survey followed a stepwise, sequential approach comprising the following stages:

- Preparation for the review
    - Question formulation
    - Search string formulation
- Collection of data
    - Bibliographic searches
    - Collation of retrieved records from databases searched through
- Selection of relevant references
    - Screening of records for relevance
        - Initial screening and disposal of irrelevant records
    - Retrieval of full references
        - In-depth screening, omission of irrelevant references
- Full-text data mining
    - Mapping of evidence
    - Critical appraisal
- Data extraction (Appendix 3) and summary of the outcomes (Chapter 4)

These steps by and large follow the methodology described elsewhere for systematic reviews, albeit that the current search will be more flexible, geared towards the comprehensive inclusion of all relevant aspects and information, rather than within the boundaries of a particular review question.

Preparation for the review

The questions that the literature survey sought to answer were the following:

1. *What are the potential food & feed safety hazards or risks linked to side effects of gene editing used for creating small mutations in crops?*
2. *What is reported in peer-reviewed literature about the nature and frequency of off-target mutations caused by genome editing tools in plants?*

These questions engendered different concepts, as follows:

- Intervention: side-effects of genetic editing of a host crop: the terms used for searching through the selected bibliographies should cover
    - the various types and synonyms for gene editing methods (e.g. CRISPR-Cas9, TALEN, ZFN) that can be applied, as well as
    - the various food & feed crops that may be modified (trivial species names, generic)
    - side effects of the gene edit (for example, off-target mutations)
- Comparator: non-edited, conventional crops currently used as food or feed with a history of safe use
- Population: Consumers (human and animal) of the edited crops
- Outcomes: health hazards and risks for consumers (for example, negative health effects, adverse reactions, toxicity, allergenicity)

Search terms

The search string was composed of sub-strings representing the different concepts described above, annealed by Boolean operators "AND" or "OR", as provided in detail in Table 1.

Table 1: *Concepts making up the components of the search string for searching bibliographic databases*

#	Concept	Search terms
1	Crops (e.g. commodity, vegetable, fruit, berry, nut, herb, spice)	abaca or agave or ajwain or alliace* or almond* or allspice or amaranth* or ambarella or angelica or anise* or annatto or apple* or apricot* or arabidopsis or areca or artichoke* or asparagus or atemoya or aubergine* or avocado* or badian or bambara or banana* or barberr* or barley or basella or basil or bay or bean* or beebrush or beet* or "bee balm" or ber or berr* or betel* or bilberr* or bilimbi or blackberr* or blueberr* or bokchoi or boldo or borage or boysenberr* or bramble* or brassica* or breadfruit* or breadnut* or brinjal or broadbean* or broccoli or buckwheat or burnet or bushberr* or cabbage* or calabash or calendula or camomile or canary* or cane or caneberr* or canella or canistel or canola or cantaloupe* or caper* or carambola or caraway or cardamom* or carob* or carom or carrot* or cashew or cashewapple or cassava or cassia or castor or catnip or cauliflower* or celery or cereal* or chamomile or chard or chataigne or chayote or cherimoya or chervil or cherr* or chestnut* or chickpea* or

		chicory or chikoo or chiku or chili or chilli* or chirimoya or chives or choi or choy or cilantaro or cilantro or cinnamon or citronella or clementine* or cloudberr* or clove* or cocoa or coconut* or cocoyam* or coffee or coir or coriander or collard* or corn or costmary or cotton* or cowpea* or cranberr* or crop* or cucumber* or cucurbit* or cumin or currant* or curry or "date palm*" or dates or dill or durian or eddo* or eggfruit* or eggplant* or elderberr* or emblic or epazote or falsa or fennel or fenugreek or feverfew or fig or figs or flax or fonio or fruit* or garlic or geranium or gherkin* or ginger or goldenberr* or gooseberr* or gourd* or grain* or grape* or groundberr* or groundnut* or guava* or gum or gums or hazelnut* or hemp or hempseed* or herb or herbs or hibiscus or hops or horsebean* or horseradish* or huckleberr* or jaboticaba or jabuticaba or jackfruit* or jambolan or jamun or jojoba or jujube or jute or kale or kapok or karite or karonda or kiwi* or kola or lablab or "lab lab" or lambsquarter* or langsat* or lanzone* or laurel or lavender or leek* or legum* or lemon* or lentil* or lettuce or licorice or lime* or lingonberr* or linseed* or litchi or longan or loquat* or lotus or lovage or lupin* or lychee* or macademia or mace or maize or mamey or mammee or mandarin* or mango* or manila or manioc or marigold or marjoram or mate or melon* or millet or mint or moringa or mulberr* or mungbean* or mushroom* or muskmelon or mustard or myrobalan or myrtle or nasturtium or nectarine* or noni or nut or nutmeg or nuts or oats or oilpalm or oilseed* or okra or olallieberr* or olive* or onion* or orange* or oregano or paddy or pakchoi or paksoi or palm or palmyra* or papaya* or paprika or parsley or parsnip* or patchouli or pawpaw or "paw paw" or pea or peach* or peanut* or pear* or peas or pecan or pepper* or perilla or persimmon* or phalsa or pickle* or pigeonpea* or pineapple* or piper or pistachio* or plant* or plantain* or plum* or pomegranate* or pomelo* or poppies or poppy or potato* or pulse* or pumpkin* or pyrethrum or quince* or quinoa or radish* or rambutan or ramie or rape* or raspberr* or redcurrant* or rhubarb or rice or ricebean* or rocket or rooibos or rose or rosehip* or rosemary or rubber or rucola or rue or rye or safflower or saffron or sage or salak or salmonberr* or sapodilla or sapot* or sassafras or satsuma* or savory or scallion or sesame or shallot* or shea or sisal or sloe* or sorghum or sorrel or soursop* or soy or soya* or soybean* or spearmint or spice* or spinach or sprout* or squash or stevia or strawberr* or stringbean* or sugar* or sumac or sunflower* or sweetpotato* or sweetsop* or tallow or tamarind or tangerine* or tansy or tapioca or taro or tarragon or tayberr* or tea or thaliana or thyme* or tinda or tobacco or tomato* or triticale or truffle* or tung or turmeric or turnip* or valerian or vanilla or vegetable* or verbena or vetch* or vetiver or vine* or walnut* or wasabi or watercress or watermelon* or wheat or wineberr* or woodruff or wormwood or yam or yams or yautia or yerba or zapot* or zucchini
2	Gene editing	Cas9 or Cas13 or chimeraplast* or "chimeric RNA" or Cpf1 or CRISPR* or crRNA or dCas9 or "directed muta*" or "directed nuclease*" or "effector nuclease" or endonuclease* or "epigenome edit*" or "gen* edit*" or "gen* technolog*" or gRNA or "guide RNA" or HDR or HITI or "homologous recombination" or "homology depend*" or "homology directed" or "homology independent" or meganuclease* or "microhomology assisted" or "microhomology mediated" or MMEJ or NHEJ or "non-homologous end-joining" or nuclease or ODM or RTDS or scissor* or SDN* or sgRNA or SpCas9 or "specific nuclease*" or SpyCas9 or SSA or SSN* or ssODN* or "strand annealing" or TALEN* or "targeted muta*" or tracRNA or ZFN* or "zinc finger"
3	Side effects of gene editing	amber or break* or delet* or diminut* or duplicat* or epigen* or eliminat* or frameshift* or imbalance* or indel* or insert* or inver* or mutation* or "number varia*" or "off target" or problem* or rearrange* or SNP* or SNV* or "structural varia*" or substitut* or translocat* or unanticipated or uninten* or unexpect* or unwant*
4	Health impacts (hazards/risks)	advers* or allerg* or anaphyla* or biohazard* or biosafe* or concern* or danger* or deleterious or deteriorat* or detriment* or fatal* or harm* or hazard* or lethal* or mortal* or poison* or risk* or safe* or security or toxic* or unhealthy

The occurrence of these terms in keywords, abstract and title (not full-text) was particularly searched for. After retrieving the records for each concept (sub-string), these were combined subsequently by combining them with the Boolean operator “AND” during the same search session within the particular bibliography being consulted, *i.e. “*#1 AND #2 AND #3 AND #4”.

Benchmarking

For refining and focusing the search strings towards optimum coverage of relevant outputs, benchmark sets were construed of relevant references for that particular substring (e.g. gene editing

in plants) and for their combination (health impacts of side effects of gene editing in crops). In case of incomplete coverage, the cause of this incompleteness will be verified (e.g. reference missing from bibliographies) and search strings adapted, if needed.

Bibliographies

Scientific bibliographies

A major factor for choosing particular databases is their coverage of the fields of life sciences, agriculture and biotechnology, as well as the comprehensiveness of the portfolio of peer-reviewed journals. Besides scientific literature in peer-reviewed journals, the various databases also provide good coverage of e.g. conference proceedings, international organizations' reports, and trade journals. The following scientific bibliographies were selected for searching for relevant records.

- Web of Science, published by Clarivate Analytics, which is specialized in scientific citations. It covers a diverse range of disciplines, including natural and life sciences, social science, and humanities. The databases included in the "all databases" search option is subscription-dependent and in this case comprised the Web of Science Core Collection, Current Contents Connect, KCI-Korean Journal Database, MEDLINE®, Russian Science Citation Index, and SciELO.
- CAB Abstracts published by the Commonwealth Agricultural Bureaux International (CABI) covers a broad range of journals and other publications published in 50 languages on the topics of agriculture, aquaculture, veterinary sciences, plant sciences, environmental sciences, microbiology & parasitology, food, nutrition, health, applied economics, and leisure & tourism
- Scopus is Elsevier's abstract and citation database, covering more than 34,000 scientific journals from thousands of publishers in the life, health, physical, and social sciences

Grey literature

Opinions from international risk assessment bodies specialized in the food/feed safety assessment of new and gene-edited/genetically modified crops were scanned for information on potential side

effects of gene editing identified in gene-edited plants evaluated by these agencies. Moreover, it was checked if and which possible consequences of these effects for health and safety of food and feeds produced from these crops have been assessed by these agencies.

Collection of data

As reference manager software for local desktop storage and processing of bibliographic records, the Endnote program (version 7 or 8) from Clarivate Analytics was used. The bibliographic searches were performed using the search strings described above in each of the three databases. These strings were adapted to the formats used in the specific databases, e.g. correct use of brackets, quotation marks, and identifiers. The output from each database were stored in a separate Endnote file. The settings were adjusted to include only the 5 most recent years of publication given that the developments are progressing fast and that developments in CRISPR-Cas9 technology started to evolve after the year 2012. From each of the three libraries with database outputs, records were exported to a new library combining these records. After automatic de-duplication, remaining duplicates were removed manually. The latter was facilitated by ordering the records according to different criteria, such as author name, title, journal, volume, pages etc. so that duplicates were easily discerned and removed. This single Endnote library file with combined and de-duplicated records served as further basis for initial screening for relevant records.

The initial screening; a selection of relevant references

The de-duplicated, combined records were screened initially for relevance based on the title and abstract of each record. The following criteria were applied:

1. The paper is about gene editing (yes/no/unclear)
    a. No -> record is removed
    b. Yes / uncertain -> record is retained
2. The paper is about gene editing in food-producing plants or animals
    a. No -> record is removed

    b. Yes / uncertain -> record is retained

3. The paper deals with the side effects of gene editing
    a. No -> record is removed
    b. Yes / uncertain -> record is retained
4. Potential safety impacts (hazards/risks) of these side effects are discussed
    a. No -> record is removed
    b. Yes / uncertain -> record is retained

Three experts went through all records and compare the outcomes of their exercise. For example, they compared the records for which there was no consensus outcome and established how the criteria above were to be applied in a consistent manner and agreed on whether to retain or omit the particular records. For the records retained, the full text versions of the cited documents were obtained for the next step.

Full-text screening and data extraction

A total of 107 papers were selected for the full-text screening method. Full-text versions of the papers were gone through in a joint action of both screening the papers for relevance based on the same above criteria for initial screening, as well as for additional mapping questions to be formulated in course of the research based on experiences gathered.

The questions that were used for this data extraction, include the following:

5. Which particular type of organism is edited (multiple answers possible)
    a. Plant: Yes (please specify) / No
    b. Animal: Yes (please specify) / No
    c. Other: Yes (please specify) / No
6. Which gene editing method was used
    a. CRISPR Cas9, Cpf12, other versions
    b. TALEN

    c. ZFN
    d. Others (specify)
7. What kind of mutation was targeted
    a. NHEJ
    b. Targeted mutations (template-directed)
    c. Epigenetic (base methylation)
    d. Other (specify)
8. What kind of molecular side effects are described
    a. Off-target DNA mutations
    b. On-target, additional DNA mutations
    c. Others (specify)
9. Specific nature of molecular side effects
    a. Amber mutations
    b. Frameshift mutations
    c. Single-nucleotide variances/polymorphisms
    d. Inversion
    e. Duplication
    f. Other rearrangements
    g. Chromosome translocation
10. Hazards/risks related to off-target effects
    a. Changed toxicity
    b. Altered allergenicity
    c. Nutritional value impacted

The answers to these questions were collected in a jointly used Excel spreadsheet, which facilitated comparison of outcomes between different reviewers, as well as the quantitative analyses of reviewers' answers to the various questions. To ensure consistency amongst reviewers, a sample of

10% (minimally 10) of the references were gone through by all reviewers, which were discussed amongst each other conflicting outcomes (at the inclusion level: yes/no; and answers to each mapping question).

Snowballing

The snowballing approach was used to search for relevant literature to answer the second question. Literature reviews from our first search were used to select relevant papers. Plant studies were first screened for a description of an off-target analysis in their results, materials and methods section or the supplementary information. The output references from the snowballing approach were stored in an Endnote file. Two experts went through all references and summarized the results in Annex 3. The outcomes of data extraction were used for a narrative description of the outcomes.

Critical appraisal of the papers

Papers that do not comply with quality / ethical criteria, such as the following ones, were discarded from the selection of relevant references:

- Data lacking on e.g. methodology
- Theoretical exercises and inferences not supported with experimental data
- Papers withdrawn from publication
- Papers with indications of obvious errors, lack of peer-review and such
- Unethical research
- Human applications of gene editing
