# Supplementary material for: Occurrence and Nature of Off-Target Modifications by CRISPR-Cas Genome Editing in Plants
Source: ACS Agric Sci Technol. 2022 Mar 3;2(2):192–201. doi: 10.1021/acsagscitech.1c00270 (PMC9075866; doi:10.1021/acsagscitech.1c00270)
Supplement: Supplementary file 2 — as1c00270_si_002.pdf [file as1c00270_si_002.pdf]

Supporting Information: Overview of the scientific literature that report the identification of potential off-target sites

Occurrence and nature of off-target modifications by CRISPR-Cas genome editing in plants

Mark H.J. Sturme[1*#], Jan Pieter van der Berg[1*], Lianne M.S. Bouwman[1], Adinda De Schrijver[2], Ruud A. de Maagd[3], Gijs A. Kleter[1], Evy Battaglia-de Wilde[1]

[1] Wageningen Food Safety Research, P.O. Box 230, 6700 AE Wageningen, The Netherlands

[2] Sciensano, Rue Juliette Wytsmanstraat 14, 1050 Brussels, Belgium

[3] Wageningen Plant Research, P.O. Box 16, 6700 AA Wageningen, The Netherlands

[*] Equal contribution

# Corresponding author: mark.sturme@wur.nl

Supplementary Table S2. Overview of the scientific literature that report the identification of potential off-target sites

This Annex includes the data from the 28 peer-reviewed papers presented in Table 1, see main text of this study.

**Plant species**	**CRISPR tool**	**Delivery and expression of components of the CRISPR system**	**Target gene(s)**	**Biased (B) or unbiased (U)**	**Description of method(s) to identify potential off-target modifications**	***In silico* prediction tool**	**Selection parameters *in silico* prediction tool**	**Off-target modifications found? Yes (Y) or no (N)**	**Number of selected off-target sites analysed**	**Number of plants analysed**	**Main off-target modification**	**Reference**
**Apple (*Malus domestica*)**	CRISPR/Cas9	genomic integration of CRISPR/Cas components through the Agrobacterium-mediated transformation	*DIPM4*	B	*in silico* prediction followed by PCR amplification and sequencing	CRISPOR	≤4 bp mismatches	N	5 potential off-target sites	6 lines	no off-target modifications were detected	(Pompili *et al.*, 2020)
**Apple (*Malus domestica*), Pear (*Pyrus communis*)**	CRISPR/Cas9	genomic integration of CRISPR/Cas components through the Agrobacterium tumefaciens - mediated transformation	*MdPDS, MdTFL1.1, PcTFL1.1*	B	*in silico* prediction followed by PCR amplification and sequencing	CRISPOR	not mentioned, refers to Haeussler et al 2016	Y	only 4 of 6 identified off-target sites analysed, only for the TFL1.1 gRNAs	5 different transgenic lines	small indels <10 bp	(Charrier *et al.*, 2019)

Arabidopsis thaliana	CRISPR/Cas9	genomic integration of CRISPR/Cas components through the Agrobacterium tumefaciens -mediated transformation	*PDX1.2*	B	*in silico* prediction followed by PCR amplification and sequencing	CRISPRdirect	not mentioned	not mentioned	4, 2 for each gRNA	not mentioned	not mentioned	(Dell'Aglio *et al.*, 2019)
	CRISPR/Cas9	genomic integration of CRISPR/Cas components through the Agrobacterium-mediated transformation	*miR160A*	B	*in silico* prediction of off-target sites	Rgenome	not mentioned	not mentioned	not mentioned	not mentioned	not mentioned	(Ouyang, Ma and Li, 2020)
	CRISPR/Cas9	genomic integration of CRISPR/Cas components through the Agrobacterium-mediated transformation	*FWA*	U	whole genome bisulfite sequencing (WGBS) to screen DNA methylation	not mentioned	not mentioned	Y	ChIP-seq showed one major off-target site	not mentioned	off-target methylation observed around target site	(Papikian *et al.*, 2019)
	CRISPR/Cas9	genomic integration of CRISPR/Cas components through the Agrobacterium-mediated transformation	*gfp*	B	*in silico* prediction followed by PCR/RE assay	CRISTA	not mentioned	N	4 potential off-target sites	not mentioned	no off-target modifications were detected	(Permyakova *et al.*, 2019)
	CRISPR/Cas9	genomic integration of CRISPR/Cas components through the Agrobacterium-mediated transformation	*TRY, CPC, PDS1, PDS2*	B	off-target analysis using Digenome-seq and targeted amplification deep sequencing of potential off-target sites	Cas-OFFinder	not mentioned	Y	22 potential off-target sites	20 mutants	indels	(Wenjie Xu *et al.*, 2019)
Arabidopsis thaliana and lettuce (Lactuca sativa)	CRISPR/Cas9	DNA-free editing; protoplast transformation of preassembled Cas9 protein-gRNA ribonucleoproteins (RNPs)	*PHYB and BRI1 gene for Arabidopsis and the lettuce BIN2 gene*	B	*in silico* prediction followed by PCR amplification and Sanger sequencing	Cas-OFFinder	up to 5 nt mismatches were allowed	N	3 selected homologous sites for PHYB, 6 for BRI1-TS1 and 4 for BRI1-TS2 that differed from the on-target site by 2-5 nt. 91 homologous sites that differed by 1–5 nt from the on-target site	3 BIN2-mutated T1 plantlets	no off-target modifications were found	(Woo *et al.*, 2015)

Arabidopsis thaliana, Nicotiana benthamiana* and *Solanum lycopersicum	SpCas9, AsCas12a and LbCas12a	genomic integration of CRISPR/Cas components through the Agrobacterium tumefaciens - mediated transformation	*TFL1, FT, XT2A, XT2B, XT1, RUBISCO, CBP*	U & B	WGS (only in *A. thaliana* DsRED mutants), *in silico* prediction tool used to specifically screen predicted off-target sites in the sequenced genome	Cas-OFFinder	up to 4nt mismatches were allowed	N	3 off-target sites	8 T2 plants for the biased analysis, as well as 2 T1 references and a pool of 5 wild type plants	No SNP, SV or Indels detected at predicted off-target sites, however WGS revealed few putative "new" mutations as well as structural variations around the target site associated with Cas12a	(Bernabé-Orts *et al.*, 2019)
**Banana and plantain (*Musa spp.*)**	CRISPR/Cas9	genomic integration of CRISPR/Cas components through the Agrobacterium-mediated transformation	*PDS*	B	*in silico* prediction followed by PCR amplification and sequencing	Breaking-Cas, BLASTN	not mentioned	N	4 potential off-target sites	5 edited events	no off-target modifications were detected	(Ntui, Tripathi and Tripathi, 2020)
**Barley (*Hordeum vulgare*)**	CRISPR/Cas9	Genomic integration of CRISPR/Cas9 components through *Agrobacterium tumefaciens*-mediated transformation of Cas9 and sgRNA vectors	*HvPM19-1 and HvPM19-3, HvPM19-1 has three close homologs: HvPM19-2, HvPM19-3 and HvPM19-4*	B	*in silico* prediction followed by PCR/Sanger sequencing	BLASTN	not mentioned	Y	2 sites were sequenced (*HvPM19-3* and *HvPM19-4*). 2 sites were sequenced (HvPM19-3 and HvPM19-4)	93 and 95 T1 plants derived from two T0 plants (T0-181 and T0-122) in which *HvPM19-1 was targeted. 73 T1 plants derived from T0-211 plants in which HvPM19-1 was targeted*	3 T0-181 progeny plants from 72 tested (4.2 %) had off-target modifications in the *HvPM19-3* gene (Indels). no off-target modifications found in the other two selected genes	(Lawrenson *et al.*, 2015)
Brassica campestris	CRISPR/Cas9	genomic integration of CRISPR/Cas components through the Agrobacterium-mediated transformation	*Bra003491, Bra007665, Bra014410*	B	*in silico* prediction followed by PCR amplification and sequencing	CRISPR-P	<2 bp mismatches	N	3 potential off-target sites	14 lines	no off-target modifications were detected	(Xiong *et al.*, 2019)
Brassica napus	CRISPR/Cas9	genomic integration of CRISPR/Cas components through the Agrobacterium-	*TT8*	B	*in silico* prediction followed by PCR amplification and sequencing	CRISPR-P	not mentioned	N	26 potential off-target sites	30 T0 gene edites plants	no off-target modifications were detected	(Zhai *et al.*, 2019)

		mediated transformation										
**Cacao (*Theobroma cacao*)**	CRISPR/Cas9	*Agrobacterium tumefaciens*-mediated transient expression of CRISPR/Cas9 components (cacao leaf infiltration) and CRISPR/Cas9 components integrated in the genome through transformation of secondary somatic embryo cotyledons	*TcNPR3* using 2 differents gRNAs	B	*in silico* prediction followed by PCR amplification and sequencing	Geneious	up to 7 nt mismatches were allowed	N	9 off-target sites for both gRNA1 and gRNA2, with 3-7 nt mismatches	10 leaf samples or 5 mutant embryos	Analysis of off-target mutagenesis in sequences similar to sgRNA target sites using high-throughput sequencing did not reveal mutations above background sequencing error rates. No off-target modifications were found in the mutant embryos.	(Fister *et al.*, 2018)
**Carrizo Citrange citrus (*Poncirus trifoliata L. Raf.* × *Citrus sinensis L. Osb.*)**	CRISPR/Cas9	Genomic integration of CRISPR/Cas9 components (vectors) through *Agrobacterium tumefaciens*-mediated transformation of gRNA/Cas9 expression vectors	Integration of construct containing hSpCas9 and eGFP encoding genes into *CsPDS* locus	B	*in silico* prediction followed by PCR amplification and sequencing	CRISPR-P	not mentioned	N	1 site for each gRNA	4 plants, 2 for each gRNA	no off-target modifications were detected	(F. Zhang *et al.*, 2017)
**Cassava (*Manihot esculenta*)**	CRISPR/Cas9	Genomic integration of CRISPR/Cas9 components (vectors) through *Agrobacterium tumefaciens*-mediated transformation of gRNA/Cas9 expression vectors	*MePDS,* by use of two different gRNAs	B	PCR amplification of 504 bp of the target sequence followed by Sanger sequencing	not applicable	not applicable	Y	only 504 bp of the target gene	11 T0 plants	substitutions and indels observed upstream of the 5′ and or downstream of the 3′ target site. Larger deletions (101 bp) were also observed	(Odipio *et al.*, 2017)
**Cassava plant (*Manihot esculenta*)**	CRISPR/Cas9	genomic integration of CRISPR/Cas components through the Agrobacterium tumefaciens -mediated transformation	*eLF4E* & isoforms	B	*in silico* prediction followed by PCR amplification and sequencing	CasOT	default CasOT settings	Y	8 potential off-target sites	3 cassava lines	indels of ≤11 bp	(Gomez *et al.*, 2019)

**Cotton (*Gossypium hirsutum*)**	CRISPR/Cas9	genomic integration of CRISPR/Cas components through the Agrobacterium-mediated transformation	*AP2, MYB44, ARC*	U	Whole genome sequencing, assessment of off-target mutations at predicted potential off-target sites	BatMis, Cas-OFFinder, CRISPR-P	≤5 bp mismatches	Y	4413 off-target sites with canonical PAMs (NGG, NAG, NGA)	14 Cas9-edited plants	Mainly SNPs and 1-4 bp indels	(J. Li *et al.*, 2019)
	CRISPR/Cas9 GhBE3 base editor	genomic integration of CRISPR/Cas components through the Agrobacterium-mediated transformation	*CLA, PEBP*	U	Whole genome sequencing as well as targeted deep sequencing of potential off-target sites	BatMis, Cas-OFFinder, CRISPR-P	Deep sequencing: ≤5 bp mismatches WGS: "high" off-score, C sites in editing window and located in coding regions	Y	~500-1000 potential off-target sites per gRNA	two based edited plants analysed by WGS	SNPs	(Qin *et al.*, 2020)
**Cowpea (*Vigna unguiculata*)**	CRISPR/Cas9	genomic integration of CRISPR/Cas components through the Agrobacterium-mediated transformation	*VuSYMRK*	B	*in silico* prediction followed by PCR amplification and sequencing	CRISPR-P	not mentioned	N	9 potential off-target sites, 3 for each gRNA	4 selected edited lines	no off-target modifications were detected	(Ji *et al.*, 2019)
**Cucumber (*Cucumis sativus*)**	CRISPR/Cas9	Genomic integration of CRISPR/Cas9 components (vectors) through *Agrobacterium tumefaciens*-mediated transformation	*eIF4E*	B	*in silico* prediction followed by PCR amplification and sequencing	CRISPR-P	off-target sites have 3-4 nt mismatches	N	5 sites	non-transgenic T3 plants (amount not mentioned)	no off-target modifications were detected	(Chandrasekaran *et al.*, 2016)
**Duncan grapefruit (*Citrus paradisi*)**	CRISPR/Cas9	Genomic integration of CRISPR/Cas9 components (vectors) through *Agrobacterium tumefaciens*-mediated transformation of epicotyl explants	*CsLOB1*	B	*in silico* prediction followed by PCR amplification and sequencing	CRISPR-P	not mentioned	N	7 sites	6 plants	no off-target modifications were detected	(Jia *et al.*, 2017)
	CRISPR/Cas12a	genomic integration of CRISPR/Cas components through the	*CsPDS, CsLOBP*	B	*in silico* prediction	Cas-OFFinder	≤2 bp mismatch with target site, RNA bulge of 1 bp	N	No potential off-target sites found with in silico prediction settings used	none	No predicted off-target sites	(Jia, Orbović and Wang, 2019)

		Agrobacterium-mediated transformation										
**Grape (*Vitis vinifera* L.)**	CRISPR/Cas9	genomic integration of CRISPR/Cas9 components through *Agrobacterium tumefaciens*-mediated transformation, using somatic embryos	*VvWRKY52* using 4 different gRNAs	B	*in silico* prediction followed by PCR amplification and sequencing	CRISPR-P	not mentioned	N	6 off-target sites, 1 site for gRNA2 and gRNA4. And 2 sites for gRNA1 and gRNA3	12 T0 plants	no off-target modifications were detected	(X. Wang *et al.*, 2018)
	CRISPR/Cas9	Genomic integration of CRISPR/Cas9 components (vectors) through *Agrobacterium tumefaciens*-mediated transformation of gRNA/Cas9 expression vectors	*IdnDH,* by use of different 2 gRNAs	B	*in silico* prediction followed by PCR amplification and Sanger sequencing	CRISPR-P	not mentioned	N	2 sites for each gRNA	10 CMs (cell mass) for gRNA1 and 3 CMs for gRNA2	no off-target modification were detected	(Ren *et al.*, 2016)
**Hongkong kumquat (*Fortunella hindsii*)**	CRISPR/Cas9	genomic integration of CRISPR/Cas components through the Agrobacterium-mediated transformation	*CCD4b, PDS*	B	*in silico* prediction followed by PCR amplification and sequencing	CRISPR-P	they mention: off-score > 0.09 as criterion	N	CCD4b: 2 potential off-target sites for gRNA1 and 3 for gRNA2. PDS: 1 potential off-target site for gRNA1 and 2 for gRNA2	2 T0 plants and 6 T1 plants for CCD4b and 5 T0 plants for PDS	no off-target modifications were detected	(C. Zhu *et al.*, 2019)
**Kiwifruit (*Actinidia* Lindl.)**	CRISPR/Cas9	genomic integration of CRISPR/Cas9 components through *Agrobacterium tumefaciens*-mediated transformation using kiwi leaf discs	*AcPDS* using 4 different gRNAs	B	*in silico* prediction followed by PCR amplification and sequencing	Cas-OFFinder	not mentioned	N	4 off-target sites, one site for each gRNA	78 callus lines	no off-target modifications were detected	(Z. Wang *et al.*, 2018)
**Maize (*Zea mays*)**	CRISPR/Cas9	Three methods tested: DNA-free (using RNPs and particle gun (PG)), and DNA-based delivery using *Agrobacterium* or PG.	Ms26 (M1 gRNA), Ms45 (M2 gRNA) and Lig1 (M3 gRNA)	B	a three-step approach:<br>1) computational prediction by Cas-OFFinder<br>2) combination of Cas-OFFinder predictions with CLEAVE-seq (a genome-wide biochemical assay)	Cas-OFFinder	up to 5 nt and 2 DNA/gRNA bulges were allowed	Y	15 sites for M1 gRNA, 95 sites for M2 gRNA and 6 sites for M3 gRNA	~300 T0 plants for the M2 gDNA and ~50 T0 plants for both the M1 and M3 gRNA	indels found in 3 predicted sites for M2	(Young *et al.*, 2019)

					data for off-target site discovery<br>3) off-target site monitoring using Molecular Inversion Probe (MIP) analysis							
	CRISPR/Cas9	Delivery of either CRISPR-Cas9 components in DNA vertors or Cas9–gRNA RNP complexes into maize immature embryo cells with gold particle borbardment	*LIG, ALS2, MS26 and MS45*	B	*in silico* prediction followed by PCR and deep sequencing	the sequence alignment software Bowtie sequence aligner	up to 2 mismatches were allowed and presence of PAM (NGG) sequence	Y	1 site	DNA delivery method: 940 T0 plants (23 out of 940 plants with off-target modifcations in the MS45 off-target site. RNP delivery method: 1880 T0 plants (0 off-target modifications found in the MS45 off-target site	indels found in the MS45 off-target site with the DNA delivery method	(Svitashev *et al.*, 2016)
	CRISPR/Cas9	genomic integration of CRISPR/Cas components through the Agrobacterium tumefaciens - mediated transformation	28 different maize genes	B	*in silico* prediction followed by PCR amplification and sequencing	WU-Blast	≤3 nt mismatch in last 15 nt with a perfect NGG PAM match, or when NGG PAM not conserved only alignments with a perfect match on the last 15 nt	N	3 potential off-target sites for each of the 28 targets	not mentioned	no off-target modifications were detected	(Doll *et al.*, 2019)
	CRISPR/Cas9 and Cas12	genomic integration of CRISPR/Cas components through the Agrobacterium-mediated transformation	*Zmgl2*	B	*in vitro* identification of off-target sites using CIRCLE-seq and TIDE combined with *in silico* prediction using Cas-OFFinder, both followed by PCR	Cas-OFFinder	≤4 bp mismatches	N	17 potential off-target sites for gRNA1 and 20 for gRNA2	56 T1 lines in total	no off-target modifications were detected	(Lee *et al.*, 2019)

					amplification and sequencing							
**Maize (*Zea mays*), rice (*Oryza sativa*) and wheat (*Triticum aestivum*)**	Cas9-PBE (plant base editor)	Both *Agrobacterium*-mediated transformation and particle bombardment were used to deliver Cas9-PBE and constructs and generate base-edited plants	*OsCDC48*, *OsNRT1.1B* and *OsSPL14* for rice, *TaLOX2* for wheat and *ZmCENH3*for maize	B	*in silico* prediction followed by PCR amplification and sequencing	Cas-OFFinder	not mentioned	N	5 potential off-target sites, each containing 3 nucleatide mismatches. Only performed for *OsCDC48*	40 pH-nCas9-PBE-induced rice mutants of *OsCDC48*	no off-target modifications were detected	(Zong *et al.*, 2017)
**Melon (*Cucumis melo*)**	CRISPR/Cas9	genomic integration of CRISPR/Cas components through the Agrobacterium tumefaciens - mediated transformation	*CmPDS*	B	*in silico* prediction followed by PCR amplification and sequencing	CRISPR-OFFinder	0-2 mismatches	N	not mentioned	not mentioned	no off-target modifications were detected	(Hooghvorst, López-Cristoffanini and Nogués, 2019)
Nicotiana benthamian a	CRISPR/Cas9	genomic integration of CRISPR/Cas components through the Agrobacterium-mediated transformation	*RDR6*	B	*in silico* prediction followed by PCR amplification and sequencing	CRISPRdire ct	not mentioned	N	2 potential off-target sites per gRNA	not mentioned	no off-target modifications were detected	(Matsuo and Atsumi, 2019)
**Octoploid strawberry (*Fragaria × ananassa*)**	CRISPR/Cas9	genomic integration of CRISPR/Cas components through the Agrobacterium-mediated transformation	*FveTm6*	B	*in silico* prediction followed by PCR amplification and sequencing	CRISPOR	not mentioned	N	2 potential off-target sites	4 plants	no off-target modifications were detected	(Martín-Pizarro, Triviño and Posé, 2019)
**Oilseed rape (*Brassica napus*)**	CRISPR/Cas9	Genomic integration of CRISPR/Cas9 components through *Agrobacterium tumefaciens*-mediated transformation of Cas9 and gRNA vectors	BnaA.ALC.a and BnaC.ALC.a (by use of only one gRNA)	B	*In silico* prediction, PCR amplification and Sanger sequencing<br><br>Extra:<br>WGS (Illumina sequencing) of one T1 plant to search for T-DNA insertions	BLASTN	not mentioned	N	2 sites	1 T1 plant and 5 T2 plants	vector backbone fragment insertions at 5 different genomic locations in the T0 plant	(Braatz *et al.*, 2017)
	CRISPR/Cas9	genomic integration of CRISPR/Cas	*BnaA9.WRKY47*	B	*in silico* prediction followed by PCR	CRISPR-P	not mentioned	N	3 potential off-target sites	9 plants	no off-target modifications were detected	(Feng *et al.*, 2020)

		components through the Agrobacterium tumefaciens - mediated transformation			amplification and sequencing							
**Plantain (*Musa sp.*)**	CRISPR/Cas9	genomic integration of CRISPR/Cas components through the Agrobacterium-mediated transformation	*eBSOLV*	B	*in silico* prediction followed by PCR amplification and sequencing	Breaking-Cas	not mentioned	Y	7 potential off-target sites	9 edited events	SNPs	(Tripathi *et al.*, 2019)
**Potato (*Solanum tuberosum*)**	CRISPR-Cas9	*Agrobacterium tumefaciens*-mediated transient expression of CRISPR/Cas9 components	*StIAA2*	B	PCR amplification and sequencing	one sequence was selected that is identical to the target site of StIAA2	not applicable	N	1 site	6 independent mutants	no off-target modifications were detected	(Wang *et al.*, 2015)
	CRISPR/Cas9	Polyethylene glycol mediated transformation of CRISPR/Cas components	*GBSS*	B	*in silico* prediction of off-target sites, comparison of different prediction tools	SSC, CHOPCHOP CRISPRater, CRISPR-P, CRISPOR	not mentioned	N	Potential off-target sites predicted, but not further analysed	none	not performed	(Johansen *et al.*, 2019)
	CRISPR/Cas9	genomic integration of CRISPR/Cas components through the Agrobacterium-mediated transformation	*GBSSI*	B	mentiones off-target sites, but performs no analysis	CRISPOR for gRNA design	<2 bp mismatches, no mismatch in seed region	not mention ed	not mentioned	not mentioned	not mentioned	(Veillet, Chauvin, *et al.*, 2019)
**Rapeseed (*Brassica napus*)**	CRISPR/Cas9	Genomic integration of CRISPR/Cas9 components (vectors) through *Agrobacterium tumefaciens*-mediated transformation of gRNA/Cas9 expression vectors	*BnaRGA*, *BnaD A2* and *BnaFUL*	B	*in silico* prediction followed by PCR amplification and sequencing	CRISPR-P	top ranking off-target sites containing fewer than 3-bp mismatches in the 12-bp seed sequence were selected	N	at least 3 off-target sites for the sgRNAs of BnaA9.RGA, BnaC9.RGA, BnaA6.RGA and BnaC7.RGA (a total of 13 putative off-target sites)	50 randomly selected T0 and T1 plants	no off-target modifications were detected	(H. Yang *et al.*, 2017)
	CRISPR/Cas9	genomic integration of CRISPR/Cas components	*LPAT2, LPAT5*	B	*in silico* prediction followed by PCR amplification and sequencing	CRISPR RGEN	<4 bp mismatch	N	14 potential off-target sites	not mentioned	no off-target modifications were detected	(K. Zhang *et al.*, 2019)

		through the Agrobacterium-mediated transformation										
	CRISPR/Cas9	genomic integration of CRISPR/Cas components through the Agrobacterium-mediated transformation	*MAX1*	B	*in silico* prediction followed by PCR amplification and sequencing	CRISPR-P	<3 bp mismatch	N	10 potential off-target sites	36 T0 plants	no off-target modifications were detected	(Zheng *et al.*, 2020)
**Rice (*Oryza sativa L. japonica*)**	CRISPR/Cas9	genomic integration of CRISPR/Cas9 components through the Agrobacterium tumefaciens -mediated transformation	*OsMIR408, OsMIR528, OsMIR815*, and *OsMIR820*, using 8 different sgRNAs	B	*in silico* prediction followed by PCR amplification and sequencing	not mentioned	not mentioned	N	5 off-target sites for each gRNA, with 1-5 nt mismatches	5 T0 plants	no off-target modifications were detected	(Zhou *et al.*, 2017)
**Rice (*Oryza sativa*)**	CRISPR/Cas9	Genomic integration of CRISPR/Cas9 components through protoplast transformation of Cas9 and either of the three different gRNA vectors	OsMPK5, by the use of three different sgRNAs (PS1, PS2 and PS3)	B	*In silico* prediction and restriction enzyme digestion suppressed PCR (RE-PCR) combined with Sanger sequencing	BLASTN	expect value and word length set to 100 and 11	Y	3 sites for PS3 (no sites were selected for PS1 and PS2)	3 T1 plants	indel in one *in silico* predicted site	(Xie and Yang, 2013)
	CRISPR/Cas9 and Cpf1	Genomic integration of CRISPR/Cas9 or Cpf1 components through *Agrobacterium tumefaciens*-mediated transformation of Cas9/Cpf1 and gRNA vectors	OsDEP1, OsLAC, OsSPL14, OsTB1, OsmiR398a, Os02circ25329, OsPDS by use of 12 different sgRNAs for Cas9 and 3 crRNAs for Cpf1	U	WGS (Illumina sequencing) complemented by an *in silico* prediction, PCR amplification and Sanger sequencing of the selected sites	Cas-OFFinder and CRISPOR	upto 10 nt mismatches were allowed	Y, only for Cas9	WGS detected off-target modifications in T0 plants editied by Cas9 for only one gRNA	26 T0 plants and 14 T1 plants edited by Cas9. 6 T0 plants and 9 T1 plants edited by Cpf1	indels found in multiple different sites for only one gRNA. No de novo off-target modifications observed in the T1 lines	(Tang *et al.*, 2019)
	CRISPR/Cas9	Genomic integration of CRISPR/Cas9 components through Agrobacterium tumefaciens-mediated transformation of Cas9 and gRNA vectors	*OsMSH1, OsDERF1, OsPDS, OsPMS3, OsMYB1, OsYSA, OsEPSPS OsMYB5, OsROC5, OsSPP*	B	*in silico* prediction followed by PCR amplification and sequencing	BLASTN	using the last 12 nt of the target sequence allowing ≤2 mismatches and using the whole 20 nt target sequence	Y	off-target modifications were dtected in 7 out of 72 plants at one predicted off-target site for the OsYSA gRNA	72 T0 plants for the OsYSA sgRNA. 10 T0 plants plus 10 T1 plants for either the OsDERF1 and OSMYB1 gRNA	indel at *in silico* predicted off-target site with 1 nt mismatch	(Zhang *et al.*, 2014)

							with E value ≤10					
	CRISPR/Cpf1	Genomic integration of CRISPR/Cpf1 components (vectors) through *Agrobacterium tumefaciens*-mediated transformation of gRNA/Cpf1 expression vectors	*OsPDS, OsBEL*	B	*in silico* prediction followed by PCR amplification and sequencing	Cas-OFFinder	homologous sequences with ≤7-bp mismatches were selected	N	19 off-target sites (9 for *OsPDS* and 10 for *OsBEL*)	For each target 5 randomly slected mutant lines were tested	no off-target modifications were detected	(Xu *et al.*, 2017)
	CRISPR/Cpf1	Genomic integration of CRISPR/Cpf1 components (vectors) through *Agrobacterium tumefaciens*-mediated transformation of gRNA/Cpf1 expression vectors	*OsEPFL9*	B	*in silico* prediction followed by PCR amplification and sequencing	Cas-OFFinder	Several paramater combinations with; ≤3-bp mismatch, ≤2-bp DNA bulge size, ≤2-bp RNA bulge size	N	10 loci with the highest off-target potential were selected	2 plants	no off-target modifications were detected	(Yin *et al.*, 2017)
	CRISPR/Cas9	Genomic integration of CRISPR/Cas9 components (vectors) through *Agrobacterium tumefaciens*-mediated transformation of gRNA/Cas9 expression vectors as well as transient expression by transformation of protoplast with gRNA and Cas9 protein	*OsBADH2, OsDEP1, OsGn1a, OsQTL, OsGS3, OsGW2, OsHd1, OsEP3 and OsLPA1*	B	*in silico* prediction followed by PCR amplification and sequencing	BLAST	not mentioned	Y	3 potential off-target sites were selected (1 for BADH2, DEP1 and EP3 gRNA)	36 plants	Off-target mutations detected in DEP1 in 4 plants and in EP3 in 2 plants	(Shen *et al.*, 2017)
	CRISPR/Cas9	genomic integration of CRISPR/Cas components through the Agrobacterium tumefaciens - mediated transformation	*TMS5*	B	*in silico* prediction followed by PCR amplification and sequencing	BLASTN ? Not clearly mentioned	not mentioned	N	4 potential off-target sites	5 T1 plants	no off-target modifications were detected	(Barman *et al.*, 2019)
	CRISPR/Cas9	genomic integration of	*OsKO1, OsKO2, OsKO3, OsKO4,*	U	Whole genome sequencing,			N		3 OsGA20ox6 mutants	Mainly SNVs and 1 indel, however no	(Chen et al 2019)

		CRISPR/Cas components through the Agrobacterium tumefaciens - mediated transformation	*OsKO5, OsKAO, OsGA20ox1, OsGA20ox2, OsGA20ox3, OsGA20ox4, OsGA20ox5, OsGA2ox6, OsGA2ox9*		assessment of small indels and SNVs most likely to be true positives						mutations found in potential off-target sites or within 100 bp of a potential off-target site as predicted by sequence homology	
	CRISPR/Cas9	genomic integration of CRISPR/Cas components through the Agrobacterium tumefaciens - mediated transformation	*OsGA20ox2*	B	*in silico* prediction followed by PCR amplification and sequencing	CRISPR-GE	not mentioned	N	2 potential off-target sites	30 plants	no off-target modifications were detected	(Han *et al.*, 2019)
	Adenine base editing using nSpCas9 fused with ecTadA*7.10 (ABE-P1S)	genomic integration of ABE components through the Agrobacterium-mediated transformation	*OsSPL14, SLR1, OsSERK2, Tms9-1, OsNRT1.1B, OsACC1, OsDEP1, SPX-MSF2, OsSPL17, OsSPL16, OsSPL18, OsSPL13, SNB*	B	*in silico* prediction followed by PCR amplification and sequencing	CRISPR-GE	≤5 bp mismatches with target site	Y	9 potential off-target sites for sgRNA1 (*OsSPL14*), 3 for sgRNA6 (*OsACC1*), 4 for sgRNA9 (*OsSPL14/17*)	8 randomly selected base edited lines	base conversion was detected at off-target site 1 of sgRNA1	(Hua *et al.*, 2020)
	CRISPR/Cas9	genomic integration of CRISPR/Cas components through the Agrobacterium-mediated transformation	*OsNF-YC10*	B	*in silico* prediction followed by PCR amplification and sequencing	BLASTP?	not mentioned	N	One homologous gene of *OsNF-YC10*	not mentioned	no off-target modifications were detected	(Jia *et al.*, 2019)
	CRISPR/Cas9	genomic integration of CRISPR/Cas components through the Agrobacterium-mediated transformation	*OsIAA3*	U	Whole genome sequencing, assessment of small indels and SNVs most likely to be true positives	No prediction tool used	no prediction tool used	Y	Genetic variants, such as SNPs and INDELs were called, all genes of the IAA and ARF families were inspected for putative off-targets modifications. Followed by genome wide variant survey	each of 7 mutants	Indel in OsIAA23 of the screened IAA/ARF genes as well as SNPs and indels detected in the genome wide screening	(Jiang *et al.*, 2019)
	CRISPR/Cas9	genomic integration of CRISPR/Cas components through the Agrobacterium-	*OsVP1*	B	*in silico* prediction followed by PCR amplification and sequencing	Cas-OFFinder	not mentioned, refers to Bae et al 2014	not mention ed	not mentioned	not mentioned	not mentioned	(Jung *et al.*, 2019)

		mediated transformation											
	CRISPR/Cas9	genomic integration of CRISPR/Cas components through the Agrobacterium-mediated transformation	*OsPLDa1*	B	*in silico* prediction	CRISPR-P	not mentioned	not mention ed	4 potential off-target sites	not mentioned	not mentioned	(Khan *et al.*, 2019)
	CRISPR/Cas9	genomic integration of CRISPR/Cas components through the Agrobacterium-mediated transformation	*OsACS1, OsACS2*	B	*in silico* prediction followed by PCR amplification and sequencing	CRISPR-P	not mentioned	N	3 potential off-target sites for each of the two gRNAs	6 T2 mutants, 3 for each target gene	no off-target modifications were detected	(Lee et al 2019)
	CRISPR/Cas9	genomic integration of CRISPR/Cas components through the Agrobacterium-mediated transformation	*SRL1, SRL2*	B	*in silico* prediction followed by PCR amplification and sequencing	CRISPR-GE	not mentioned	N	4 potential off-target sites per gRNA	30 mutant plants	no off-target modifications were detected	(Liao *et al.*, 2019)
	CRISPR/Cas9	genomic integration of CRISPR/Cas components through the Agrobacterium-mediated transformation	*MIR156*	B	*in silico* prediction followed by PCR amplification and sequencing	mirbase database	mir156 homologs	not mention ed	not mentioned	not mentioned	not mentioned	(Miao *et al.*, 2019)
	CRISPR/Cas9	genomic integration of CRISPR/Cas components through the Agrobacterium-mediated transformation	*SWEET* gene effector-binding elements	B	*in silico* prediction of off-target sites	not mentioned	not mentioned	not mention ed	14 potential off-target sites	not mentioned	not mentioned	(Oliva *et al.*, 2019)
	CRISPR/Cas9	genomic integration of CRISPR/Cas components through the Agrobacterium-mediated transformation	*Wx*	B	*in silico* prediction followed by PCR amplification and sequencing	E-CRISP	only NGG PAM, G as 5' base, many mismatches tolerated, non-seed region & introns ignored	N	3 potential off-target sites	not mentioned	no off-target modifications were detected	(Pérez *et al.*, 2019)

	CRISPR/Cas9	genomic integration of CRISPR/Cas components through the Agrobacterium-mediated transformation	*MPK7, MPK8, MPK10, MPK1, SERK1, SERK2, ETR2, GSK4, BZR1, CERK1, Os03g02040*	B	*in silico* prediction followed by PCR amplification and sequencing	not mentioned	not mentioned	Y	3 and 2 potential off-target sites for *MPK10* and *MPK11* respectively	not mentioned	Indels in the off-target gene *MPK9*	(Q. Ren *et al.*, 2019)
	CRISPR/Cas9	genomic integration of CRISPR/Cas components through the Agrobacterium-mediated transformation	*Tos17*	B	*in silico* prediction of off-target sites	CRISPRdirect	not mentioned	not mentioned	not mentioned	not mentioned	not mentioned	(Saika *et al.*, 2019)
	CRISPR/Cas9	genomic integration of CRISPR/Cas components through the Agrobacterium-mediated transformation	*Ald5H1*	B	*in silico* prediction followed by PCR amplification and sequencing	CRISPR-P	not mentioned	N	5 potential off-target sites	4 T1 lines	no off-target modifications were detected	(Takeda *et al.*, 2019)
	CRISPR/Cas9	genomic integration of CRISPR/Cas components through the Agrobacterium-mediated transformation	*OsRR9, OsRR10*	B	analysis of off-target editing in homolog *ORR4*	not mentioned	not mentioned	N	1 potential off-target site	not mentioned	no off-target modifications were detected	(Wang *et al.*, 2019)
	CRISPR/Cas9	genomic integration of CRISPR/Cas components through the Agrobacterium-mediated transformation	*OsLHT1*	B	*in silico* prediction followed by PCR amplification and sequencing	offTarget	not mentioned	N	4 potential off-target sites	not mentioned	no off-target modifications were detected	(X. Wang *et al.*, 2019)
	CRISPR/Cas9-PmCDA1 base editor	genomic integration of CRISPR/Cas components through the Agrobacterium-mediated transformation	*OsWaxy*	B	*in silico* prediction followed by PCR amplification and sequencing	Cas-OFFinder	3-5 bp mismatches	N	10 potential off-target sites	not mentioned	no off-target modifications were detected	(Wu *et al.*, 2019)
	CRISPR/Cas9-PmCDA1 base editor	genomic integration of CRISPR/Cas components	*ALS, NRT1.1B*	B	*in silico* prediction followed by PCR amplification and sequencing	not mentioned	not mentioned	Y	3 potential off-target sites per base-editing site	8 independent transgenic calli	SNPs	(Wen Xu *et al.*, 2019)

		through the Agrobacterium-mediated transformation										
	CRISPR/Cas9 xCas9 base editor	genomic integration of CRISPR/Cas components through the Agrobacterium-mediated transformation	*GS3, DEP1, PDS*	B	*in silico* prediction followed by PCR amplification and sequencing	Cas-OFFinder, CRISPOR	not mentioned	N	5 potential off-target sites	not mentioned	no off-target modifications were detected	(Zhong et al 2019)
	CRISPR/Cas9	genomic integration of CRISPR/Cas components through the Agrobacterium-mediated transformation	*Rc*	B	*in silico* prediction followed by PCR amplification and sequencing	CRISPR-P and BLASTN	<5 mismatches	N	11 potential off-target sites	10 T1 plants	no off-target modifications were detected	(Y. Zhu *et al.*, 2019)
**Rice (*Oryza sativa*), maize (*Zea mays*), *Arabidopsis thaliana***	CRISPR/Cas12a	genomic integration of CRISPR/Cas components through the Agrobacterium-mediated transformation	*OsROC5, OsDEP1, OsPDS, AtGL2, AtTT4, AtPAP1, ZmGl2*	none, only on-target analysis	none, only on-target analysis, refers to previous WGS analysis	not mentioned	not mentioned	not mention ed	none, only on-target analysis	-	-	(Malzahn *et al.*, 2019)
**Rice (*Oryzae sativa*)**	CRISPR/FnCpf1	Genomic integration of CRISPR/FnCp1components through *Agrobacterium tumefaciens*-mediated transformation of FnCp1 and sgRNA vectors	*OsNCED1, OsAO1 and OsAO2 (one gRNA to target both AO genes)*	B	homologous gene selection and sequencing	not mentioned	not applicable	Y	9 sites, all in homologous genes	18 T1 plants	indels observed in one off-target site for *OsNCEd1* and two off-target sites for *OsAO1/OsAO2*	(Endo *et al.*, 2016)
**Rice (*Oryzae sativa*)**	CRISPR/Cas9	Genomic integration of CRISPR/Cas9 components through protoplast transformation of Cas9 and either of the three different sgRNA vectors	*OsMPK2* and *OsPDS*	B	PCR/RE assay	not mentioned	not applicable	Y	1 site with 3 base mismatches to the PDS-SP1 and 2 sites with 1 base mismatch to the MPK2	Not mentioned, pooled transformed protoplats were used for analysis	off-target modifications found in a homologous site around the on-target site, resulting in larger on-target deletion events instead of indels	(Shan *et al.*, 2013)
Rice (Oryza sativa), tomato (Solanum	CRISPR/Cas9	genomic integration of CRISPR/Cas components	*IAMT genes (At5g55250, Os04g56950,*	B	*off-target analysis mentioned*	ARES-GT	<5 nt mismatches	not mention ed			not mentioned	(Aliaga-Franco *et al.*, 2019)

lycopersicum ***),Arabidopsis thaliana***		through the Agrobacterium tumefaciens - mediated transformation	*Solyc07g64990, Solyc12g14500*)									
**Soy bean (*Glycine max*)**	CRISPR/Cas9	genomic integration of CRISPR/Cas9 components through *Agrobacterium tumefaciens*-mediated transformation	*GmFT2a* using 3 different gRNAs	B	*in silico* prediction followed by PCR amplification and sequencing	CRISPR-P	not mentioned	N	6 off-target sites, 2 sites for each gRNA	18 T1 plants for 2 off-target sites for gRNA1, 35 T1 plants for 2 off-target sites for gRNA2 and 11 T1 plants for 2 off-target sites for gRNA3. The off-target sites possessed 2-4 nt mismatches	no off-target modifications were detected	(Cai *et al.*, 2018)
	CRISPR/Cas9	Genomic integration of CRISPR/Cas9 components (vectors) through *Agrobacterium rhizogenes*-mediated transformation	*Glyma07g14530, 2 DDM1 genes (Glyma01g38150, Glyma11g07220), MET1, and miR1514*	B	*in silico* prediction followed by PCR amplification Sanger sequencing	BLASTN	up to 6 nt mismatches were allowed	Y	putative off-target sites were identified with 2-6 mismatches for the Glyma07g14530 (10 sites), 2 DDM1 genes (1 site each), MET1 (1 site), and miR1514 (2 sites) gRNAs	4 to 10 root tips for each gRNA	Two gRNAs (miR1514 and *Glyma11g07220)* created off-target modifications (indel) in one of the predicted off-target sites. 2 nt mismatches between Glyma11g07220 gRNA and off-target site Glyma01g38150. 2 nt mismatches between miR1514 and the 18g off-target site	(Jacobs *et al.*, 2015)
	CRISPR/Cas9	Genomic integration of CRISPR/Cas9 components (vectors) through Agro-bacterium rhizogenes-mediated transformation	Glyma06g14180, Glyma08g02290 and Glyma12g37050	B	*in silico* prediction followed by PCR amplification and Sanger sequencing	customized Perl scripts and the USEARCH program	not mentioned	Y	Glyma06g14180 gRNA had identical sequence in an off-target site in Glyma04g40610. Glyma08g02290 gRNA was identical to an off-target site in Glyma05g37270. 1 putative off-target site	5 out of 14 independent mutants showed a modifications at the predicted off-target site. Not tested. 2 out of 22	Two gRNAs (Glyma06g14180 and Glyma12g37050) created off-target modifications in the predicted off-target sites	(Sun *et al.*, 2015)

									(Glyma09g00490) was identified with 1 mismatches for the Glyma12g37050 gNRA	independent mutants showed a modifications at the predicted off-target site		
	CRISPR/Cas9	genomic integration of CRISPR/Cas9 components through the *Agrobacterium rhizogenes*-mediated transformation (hairy-root ex vitro transformation assay)	*GmDrb2a, GmDrb2b* using 2 different gRNAs	B	*in silico* prediction and manual screening of WGS data	CRISPR-P	not mentioned	not mentioned	19 sites for each gRNA, with 2-4 nt mismatches	2 T0 plants and 4 T1 plants	not mentioned	(Curtin *et al.*, 2018)
	CRISPR/Cas9	genomic integration of CRISPR/Cas9 components through the *Agrobacterium rhizogenes*-mediated transformation	*GmPPD1* and *GmPPD2* using one gRNA	B	*in silico* prediction followed by PCR amplification and sequencing	CRISPR-P	not mentioned	N	3 sites, with 4 nt mismatches	3 T1 plants	no off-target modifications were detected	(Kanazashi *et al.*, 2018)
	CRISPR/Cas9	genomic integration of CRISPR/Cas components through the Agrobacterium tumefaciens - mediated transformation	*FAD2-2*	B	*in silico* prediction followed by PCR amplification and sequencing	CRISPR-P	not mentioned	N	19 off-target sites	8 plants	no off-target modifications were detected	(al Amin *et al.*, 2019)
	CRISPR/Cas9	genomic integration of CRISPR/Cas components through the Agrobacterium tumefaciens - mediated transformation	*GmRIC1, GmRIC2*	B	*in silico* prediction followed by PCR amplification and sequencing	Cas-OFFinder	not mentioned	N	10 sites, 5 for each target	not mentioned	no off-target modifications were detected	(Bai *et al.*, 2020)
	CRISPR/Cas9	genomic integration of CRISPR/Cas components through the	*GmFAD2*	B	*in silico* prediction followed by PCR amplification and sequencing	CRISPR-P	not mentioned	N	2 potential off-target sites	30 T2 plants	no off-target modifications were detected	(Do *et al.*, 2019)

		Agrobacterium tumefaciens - mediated transformation										
**Sweet orange (*Citrus X sinensis*)**	CRISPR/Cas9	Agroinfiltration for transient expression of CRISPR-Cas9 components in leaves	*CsPDS*	B	*in silico* prediction followed by PCR/RE assay	BLASTN	not mentioned	N	8 sites with 4 to 7 mismatches to the sgRNA-*CsPDS*	not mentioned	no off-target modifications found	(Jia and Nian, 2014)
**Tomato (*Solanum lyco-persicum*)**	CRISPR/Cas9	Genomic integration of CRISPR/Cas9 components (vectors) through *Agrobacterium tumefaciens*-mediated transformation of gRNA/Cas9 expression vectors	*SlSGR1, SlLCY-E, SlBlc, SlLCY-B1, SlLCY-B2*	B	*in silico* prediction followed by PCR amplification and sequencing	CRISPR-P	not mentioned	N	2 potential off-target sites for each gRNA (10 total)	21 plants	no off-target modifications were detected	(Li *et al.*, 2018)
	CRISPR/Cas9	Genomic integration of CRISPR/Cas9 components (vectors) through *Agrobacterium tumefaciens*-mediated transformation of gRNA/Cas9 expression vectors	*SlALC*	B	*in silico* prediction followed by PCR amplification and sequencing	BLAST	not mentioned	N	3 potential off-target sites	30 plants from T0 and T1 transgenic plants were randomly selected	no off-target modifications were detected	(Yu *et al.*, 2017)
	CRISPR/Cas9	genomic integration of CRISPR/Cas9 components through Agrobacterium tumefaciens - mediated of cotyledons	*SlMAPK3* using 2 different gRNAs	B	*in silico* prediction followed by PCR amplification and sequencing	CRISPR-P	not mentioned	N	3 off-target sites for each gRNA	15 T1 plants (5 T1 plant of each line)	no off-target modifications were detected	(Wang *et al.*, 2017)
	CRISPR/Cas9	genomic integration of CRISPR/Cas9 components through the *Agrobacterium tumefaciens* - mediated transformation of leaf disks	*SlIAA9* using 3 different gRNAs	B	*in silico* prediction followed by PCR amplification and sequencing	CasOT	not mentioned	N	2 off-target sites for gRNA2, with 4 nt and 5 nt mismatches. And 2 off-target sites for gRNA3 with each 4 nt mismatches	not mentioned	no off-target modifications were detected	(Ueta *et al.*, 2017)

	CRISPR/Cas9	genomic integration of CRISPR/Cas9 components through the *Agrobacterium tumefaciens* - mediated transformation	SlORRM4 using four different gRNAs	B	*in silico* prediction followed by PCR amplification and sequencing	not mentioned	not mentioned	N	2 off-target sites for each gRNA	4 T0 plants for gRNA1 and 3 To plants for gRNA2 and gRNA4, with 1-4 nt mismatches	no off-target modifications were detected, but they found a large 544-bp deletion on one allele at the SlORRM4 locus (on-target effect)	(Y. Yang *et al.*, 2017)
	CRISPR/Cas9	genomic integration of CRISPR/Cas components through the Agrobacterium tumefaciens - mediated transformation	*SlCCD8*	B	*in silico* prediction followed by PCR amplification and sequencing	CRISPR-P	<4 nt mismatches with gRNA	N	3 potential off-target sites	2 plants of each line of T1 (4), 8 total	no off-target modifications were detected	(Bari *et al.*, 2019)
	CRISPR/Cas9	genomic integration of CRISPR/Cas components through the Agrobacterium-mediated transformation	*SlEIN2, SlARF2B, SlERFE1, SlGRAS8, SlACS2, SlACS4*	B	*in silico* prediction followed by PCR amplification and sequencing	CRISPR-P	not mentioned	N	4 potential off-target sites of *SlGRAS8*	5 T1 plants and 10 T4 plants	no off-target modifications were detected	(Hu *et al.*, 2019)
	CRISPR/Cas9	genomic integration of CRISPR/Cas components through the Agrobacterium-mediated transformation	*SlNPR1*	B	*in silico* prediction followed by PCR amplification and sequencing	CRISPR-GE	≤4 bp mismatches	N	3 potential off-target sites per gRNA	10 randomly selected T1 transgenic plants	no off-target modifications were detected	(R. Li *et al.*, 2019)
	CRISPR/Cas9	genomic integration of CRISPR/Cas components through the Agrobacterium-mediated transformation	*HY5*	B	*in silico* prediction of off-target sites	BLAST	not mentioned	N	None, no off-target sites found in the genome using BLAST	--	--	(Qiu *et al.*, 2019)
	CRISPR/Cas9	genomic integration of CRISPR/Cas components through the Agrobacterium-mediated transformation	*lncRNA2155*	B	*in silico* prediction followed by PCR amplification and sequencing	CRISPR-P	not mentioned	N	2 potential off-target sites	not mentioned	no off-target modifications were detected	(Yu *et al.*, 2019)

**Tomato (Solanum lyco-persicum) & potato (Solanum tuberosum)**	CRISPR/Cas9 base editor	genomic integration of CRISPR/Cas components through the Agrobacterium-mediated transformation	*ALS* genes	B	analysis of off-target editing in homolog *ALS2*	not mentioned	not mentioned	Y	1 potential off-target site	25 plants	Indels	(Veillet, Perrot, *et al.*, 2019)
**Wanjincheng orange (*Citrus sinensis* Osbeck)**	CRISPR/Cas9	Genomic integration of CRISPR/Cas9 components (vectors) through *Agrobacterium tumefaciens*-mediated transformation of gRNA/Cas9 expression vectors	EBE_{PthA4} element of *CsLOB1*	B	*in silico* prediction followed by PCR amplification and sequencing	CRISPR-P	not mentioned	Y	11 putative off-target loci containing a PAM and that showed high sequence similarity to the two sgRNA target sites	4 plants	off-target mutations were detected in all 11 predicted sites, all consisting of 1-bp point mutations	(Peng *et al.*, 2017)
	CRISPR/Cas9	genomic integration of CRISPR/Cas components through the Agrobacterium-mediated transformation	*WRKY22*	U	whole genome sequencing in conjunction with *in silico* prediction	CRISPR-P	≤4 bp mismatches	Y	6 potential off-target sites	3 mutant lines	SNPs and Indels	(L. Wang *et al.*, 2019)
**Watermelon (*Citrullus lanatus*)**	CRISPR/Cas9	Genomic integration of CRISPR/Cas9 components through protoplast transformation of Cas9 and gRNA vectors	*ClPDS,* by use of two different gRNAs	B	*in silico* prediction followed by PCR amplification and Sanger sequencing	BLASTN	not mentioned	N	1 site for gRNA1, containing 5 nt mismatches and no PAM sequence. 2 sites for gRNA2, containing 3 nt mismatches and no PAM sequence	16 plants	no off-target modification were detected	(Tian *et al.*, 2017)
	CRISPR/Cas9	Genomic integration of CRISPR/Cas9 components through protoplast transformation of Cas9 and gRNA vectors	*WIP1*	B	*in silico* prediction followed by PCR amplification and sequencing	CRISPR-P	not mentioned	not mention ed	not mentioned	not mentioned	not mentioned	(J. Zhang *et al.*, 2020)
**Wheat (*Triticum aestivum*)**	CRISPR/Cas9		*TaEDR1*	B	*In silico* prediction, PCR restriction enzyme detection of potential off-target sites	CasOT		N	7 sites with 2 to 4 nt mismatches	6 T1 plants	no off-target modifications found	(Y. Zhang *et al.*, 2017)

	CRISPR/Cas9	Transgene free gene editing, based on transient expression of CRISPR-Cas9 DNA or *in vitro* transcripts of cas9-coding sequence and guide RNA	*TaGASR7, TaDEP1, TaNAC2, TaPIN1, TaLOX2, TdGASR7, TaGW2*	B	*in silico* prediction followed by PCR-RE	CasOT	not mentioned	Y	8 sites for TaGW2-sgRNA with 3 to 4 nt mismatches, 24 sites for TaGASR7-sgRNA with 2 to 5 nt mismatches	67 T0 plants, 101 T0 plants	indels found in homologous gene *TaGW2-A1*	(Zhang *et al.*, 2016)
	CRISPR/Cas9	Biolistic transformation of wheat protoplasts with Cas9 and gRNA vectors	*EPSPS*	B	deep sequencing of homoeoalleles of the target gene	WU-CRISPR for on-target prediciton, CRISPResso for analysis	not mentioned	Y	Homoeoallele DS	3 replicates for each of 7 gRNAs	Small indels were detected and more frequently large insertions of ≥20 bp	(Arndell *et al.*, 2019)
	CRISPR/Cas9	genomic integration of CRISPR/Cas components through the Agrobacterium-mediated transformation	*MS1*	B	*in silico* prediction followed by PCR amplification and sequencing	WU-CRISPR, sgRNA designer	not mentioned	N	not mentioned	not mentioned	no off-target modifications were detected	(Okada *et al.*, 2019)
	CRISPR/Cas9	genomic integration of CRISPR/Cas components through the Agrobacterium-mediated transformation	*Pinb, DA1, DA2, NCED1, LPR2*	B	*in silico* prediction followed by PCR amplification and sequencing	CRISPRdirect, CRISPOR	<3 bp mismatch	N	6 potential off-target sites	24 T2 progeny plants	no off-target modifications were detected	(Zhang *et al.*, 2018)
	CRISPR/Cas9	genomic integration of CRISPR/Cas components through the Agrobacterium-mediated transformation	*CKX2-1, GLW7, GW2, GW8*	B	*in silico* prediction followed by PCR amplification and sequencing	MUSCLE	<4 bp mismatch	N	8 potential off-target sites	24 mutant plants	no off-target modifications were detected	(Z. Zhang *et al.*, 2019)

References

Aliaga-Franco, N. *et al.* (2019) 'Identification of Transgene-Free CRISPR-Edited Plants of Rice, Tomato, and Arabidopsis by Monitoring DsRED Fluorescence in Dry Seeds', *Frontiers in Plant Science*. Frontiers Media S.A., 10, p. 1150. doi: 10.3389/fpls.2019.01150.

al Amin, N. *et al.* (2019) 'CRISPR-Cas9 mediated targeted disruption of FAD2–2 microsomal omega-6 desaturase in soybean (*Glycine max.L*)', *BMC Biotechnology*. BioMed Central Ltd., 19(1), p. 9. doi: 10.1186/s12896-019-0501-2.

Arndell, T. *et al.* (2019) 'GRNA validation for wheat genome editing with the CRISPR-Cas9 system', *BMC Biotechnology*. BioMed Central Ltd., 19(1), p. 71. doi: 10.1186/s12896-019-0565-z.

Bai, M. *et al.* (2020) 'Generation of a multiplex mutagenesis population via pooled CRISPR-Cas9 in soya bean', *Plant Biotechnology Journal*. Blackwell Publishing Ltd, 18(3), pp. 721–731. doi: 10.1111/pbi.13239.

Bari, V. K. *et al.* (2019) 'CRISPR/Cas9-mediated mutagenesis of CAROTENOID CLEAVAGE DIOXYGENASE 8 in tomato provides resistance against the parasitic weed *Phelipanche aegyptiaca'*, *Scientific Reports*. Nature Publishing Group, 9(1). doi: 10.1038/s41598-019-47893-z.

Barman, H. N. *et al.* (2019) 'Generation of a new thermo-sensitive genic male sterile rice line by targeted mutagenesis of TMS5 gene through CRISPR/Cas9 system', *BMC Plant Biology*. BioMed Central Ltd., 19(1), pp. 1–9. doi: 10.1186/s12870-019-1715-0.

Bernabé-Orts, J. M. *et al.* (2019) 'Assessment of Cas12a-mediated gene editing efficiency in plants', *Plant Biotechnology Journal*. Blackwell Publishing Ltd, 17(10), pp. 1971–1984. doi: 10.1111/pbi.13113.

Braatz, J. *et al.* (2017) 'CRISPR-Cas9 Targeted Mutagenesis Leads to Simultaneous Modification of Different Homoeologous Gene Copies in Polyploid Oilseed Rape (*Brassica napus*).', *Plant physiology*. American Society of Plant Biologists, 174(2), pp. 935–942. doi: 10.1104/pp.17.00426.

Cai, Y. *et al.* (2018) 'CRISPR/Cas9-mediated targeted mutagenesis of *GmFT2a* delays flowering time in soya bean', *Plant Biotechnology Journal*, 16(1), pp. 176–185. doi: 10.1111/pbi.12758.

Chandrasekaran, J. *et al.* (2016) 'Development of broad virus resistance in non-transgenic cucumber using CRISPR/Cas9 technology', *Molecular plant pathology*. Blackwell Publishing Ltd, 17(7), pp. 1140–1153. doi: 10.1111/mpp.12375.

Charrier, A. *et al.* (2019) 'Efficient Targeted Mutagenesis in Apple and First Time Edition of Pear Using the CRISPR-Cas9 System', *Frontiers in Plant Science*. Frontiers Media S.A., 10, p. 40. doi: 10.3389/fpls.2019.00040.

Chen, J. S. *et al.* (2017) 'Enhanced proofreading governs CRISPR-Cas9 targeting accuracy.', *Nature*. Howard Hughes Medical Institute, 550(7676), pp. 407–410. doi: 10.1038/nature24268.

Curtin, S. J. *et al.* (2018) 'CRISPR/Cas9 and TALENs generate heritable mutations for genes involved in small RNA processing of *Glycine max* and *Medicago truncatula'*, *Plant Biotechnology Journal*. Blackwell Publishing Ltd, 16(6), pp. 1125–1137. doi: 10.1111/pbi.12857.

Dell'Aglio, E. *et al.* (2019) 'Clarification of the dispensability of PDX1.2 for Arabidopsis viability using CRISPR/Cas9', *BMC Plant Biology*. BioMed Central Ltd., 19(1), p. 464. doi: 10.1186/s12870-019-2071-9.

Do, P. T. *et al.* (2019) 'Demonstration of highly efficient dual gRNA CRISPR/Cas9 editing of the homeologous GmFAD2-1A and GmFAD2-1B genes to yield a high oleic, low linoleic and α-linolenic acid phenotype in soybean', *BMC Plant Biology*. BioMed Central Ltd., 19(1), p. 311. doi: 10.1186/s12870-019-1906-8.

Doll, N. M. *et al.* (2019) 'Single and multiple gene knockouts by CRISPR–Cas9 in maize', *Plant Cell Reports*. Springer Verlag, 38(4), pp. 487–501. doi: 10.1007/s00299-019-02378-1.

Endo, A. *et al.* (2016) 'Efficient targeted mutagenesis of rice and tobacco genomes using Cpf1 from *Francisella novicida*.', *Scientific reports*. Nature Publishing Group, 6, p. 38169. doi: 10.1038/srep38169.

Feng, Y. *et al.* (2020) 'Transcription factor BnaA9.WRKY47 contributes to the adaptation of *Brassica napus* to low boron stress by up-regulating the boric acid channel gene *BnaA3.NIP5;1*', *Plant Biotechnology Journal*. Blackwell Publishing Ltd, 18(5), pp. 1241–1254. doi: 10.1111/pbi.13288.

Fister, A. S. *et al.* (2018) 'Transient Expression of CRISPR/Cas9 Machinery Targeting TcNPR3 Enhances Defense Response in *Theobroma cacao'*, *Frontiers in Plant Science*, 9. doi: 10.3389/fpls.2018.00268.

Gomez, M. A. *et al.* (2019) 'Simultaneous CRISPR/Cas9-mediated editing of cassava *eIF4E* isoforms *nCBP-1* and *nCBP-2* reduces cassava brown streak disease symptom severity and incidence', *Plant Biotechnology Journal*. Blackwell Publishing Ltd, 17(2), pp. 421–434. doi: 10.1111/pbi.12987.

Han, Y. *et al.* (2019) 'Generation of semi-dwarf rice (*Oryza sativa L*.) lines by CRISPR/Cas9-directed mutagenesis of OsGA20ox2 and proteomic analysis of unveiled changes caused by mutations', *3 Biotech*. Springer Verlag, 9(11). doi:

10.1007/s13205-019-1919-x.

Hooghvorst, I., López-Cristoffanini, C. and Nogués, S. (2019) 'Efficient knockout of phytoene desaturase gene using CRISPR/Cas9 in melon', *Scientific Reports*. Nature Research, 9(1). doi: 10.1038/s41598-019-53710-4.

Hu, N. *et al.* (2019) 'Rapid and user-friendly open-source CRISPR/Cas9 system for single- or multi-site editing of tomato genome', *Horticulture Research*. Nature Publishing Group, 6(1), p. 7. doi: 10.1038/s41438-018-0082-6.

Hua, K. *et al.* (2020) 'Simplified adenine base editors improve adenine base editing efficiency in rice', *Plant Biotechnology Journal*. Blackwell Publishing Ltd, 18(3), pp. 770–778. doi: 10.1111/pbi.13244.

Jacobs, T. B. *et al.* (2015) 'Targeted genome modifications in soybean with CRISPR/Cas9', *BMC Biotechnology*, 15(1), p. 16. doi: 10.1186/s12896-015-0131-2.

Ji, J. *et al.* (2019) 'Genome Editing in *Cowpea Vigna* unguiculata Using CRISPR-Cas9', *International Journal of Molecular Sciences*. NLM (Medline), 20(10), p. 2471. doi: 10.3390/ijms20102471.

Jia, H. *et al.* (2017) 'Genome editing of the disease susceptibility gene CsLOB1 in citrus confers resistance to citrus canker', *Plant Biotechnology Journal*. Blackwell Publishing Ltd, 15(7), pp. 817–823. doi: 10.1111/pbi.12677.

Jia, H. and Nian, W. (2014) 'Targeted genome editing of sweet orange using Cas9/sgRNA', *PLoS ONE*. Public Library of Science, 9(4). doi: 10.1371/journal.pone.0093806.

Jia, H., Orbović, V. and Wang, N. (2019) 'CRISPR -LbCas12a-mediated modification of citrus', *Plant Biotechnology Journal*. Blackwell Publishing Ltd, 17(10), pp. 1928–1937. doi: 10.1111/pbi.13109.

Jia, S. *et al.* (2019) 'OsNF-YC10, a seed preferentially expressed gene regulates grain width by affecting cell proliferation in rice', *Plant Science*. Elsevier Ireland Ltd, 280, pp. 219–227. doi: 10.1016/j.plantsci.2018.09.021.

Jiang, M. *et al.* (2019) 'Different knockout genotypes of OsIAA23 in rice using CRISPR/Cas9 generating different phenotypes', *Plant Molecular Biology*. Springer Netherlands, 100(4–5), pp. 467–479. doi: 10.1007/s11103-019-00871-5.

Johansen, I. E. *et al.* (2019) 'High efficacy full allelic CRISPR/Cas9 gene editing in tetraploid potato', *Scientific Reports*. Nature Research, 9(1), p. 17715. doi: 10.1038/s41598-019-54126-w.

Jung, Y. J. *et al.* (2019) 'Acquisition of seed dormancy breaking in rice (*Oryza sativa L.*) via CRISPR/Cas9-targeted mutagenesis of OsVP1 gene', *Plant Biotechnology Reports*. Springer Tokyo, 13(5), pp. 511–520. doi: 10.1007/s11816-019-00580-x.

Kanazashi, Y. *et al.* (2018) 'Simultaneous site-directed mutagenesis of duplicated loci in soybean using a single guide RNA', *Plant Cell Reports*. Springer Verlag, 37(3), pp. 553–563. doi: 10.1007/s00299-018-2251-3.

Khan, M. S. S. *et al.* (2019) 'Mutational Analysis of *OsPLDα1* Reveals Its Involvement in Phytic Acid Biosynthesis in Rice Grains', *Journal of Agricultural and Food Chemistry*. American Chemical Society, 67(41), pp. 11436–11443. doi: 10.1021/acs.jafc.9b05052.

Lawrenson, T. *et al.* (2015) 'Induction of targeted, heritable mutations in barley and Brassica oleracea using RNA-guided Cas9 nuclease', *Genome Biology*. BioMed Central, 16(1), p. 258. doi: 10.1186/s13059-015-0826-7.

Lee, H. et al. (2019) 'Editing of the OsACS locus alters phosphate deficiency-induced adaptive responses in rice seedlings. Journal of Experimental Botany. 70(6). pp 1927–1940. https://doi.org/10.1093/jxb/erz074.

Lee, K. *et al.* (2019) 'Activities and specificities of CRISPR/Cas9 and Cas12a nucleases for targeted mutagenesis in maize', *Plant Biotechnology Journal*. Blackwell Publishing Ltd, 17(2), pp. 362–372. doi: 10.1111/pbi.12982.

Li, J. *et al.* (2019) 'Whole genome sequencing reveals rare off-target mutations and considerable inherent genetic or/and somaclonal variations in CRISPR/Cas9-edited cotton plants', *Plant Biotechnology Journal*. Blackwell Publishing Ltd, 17(5), pp. 858–868. doi: 10.1111/pbi.13020.

Li, R. *et al.* (2019) 'CRISPR/Cas9-Mediated SlNPR1 mutagenesis reduces tomato plant drought tolerance', *BMC Plant Biology*. BioMed Central Ltd., 19(1), p. 38. doi: 10.1186/s12870-018-1627-4.

Li, X. *et al.* (2018) 'Lycopene Is Enriched in Tomato Fruit by CRISPR/Cas9-Mediated Multiplex Genome Editing', *Frontiers in Plant Science*, 9. doi: 10.3389/fpls.2018.00559.

Liao, S. *et al.* (2019) 'CRISPR/Cas9-Induced Mutagenesis of Semi-Rolled Leaf1,2 Confers Curled Leaf Phenotype and Drought Tolerance by Influencing Protein Expression Patterns and ROS Scavenging in Rice (*Oryza sativa L.)*', *Agronomy*. MDPI AG, 9(11), p. 728. doi: 10.3390/agronomy9110728.

Malzahn, A. A. *et al.* (2019) 'Application of CRISPR-Cas12a temperature sensitivity for improved genome editing in rice, maize, and Arabidopsis', *BMC Biology*. BioMed Central Ltd., 17(1), p. 9. doi: 10.1186/s12915-019-0629-5.

Martín-Pizarro, C., Triviño, J. C. and Posé, D. (2019) ‘Functional analysis of the TM6 MADS-box gene in the octoploid strawberry by CRISPR/Cas9-directed mutagenesis’, *Journal of Experimental Botany*. Oxford University Press, 70(3), pp. 949–961. doi: 10.1093/jxb/ery400.

Matsuo, K. and Atsumi, G. (2019) ‘CRISPR/Cas9-mediated knockout of the RDR6 gene in Nicotiana benthamiana for efficient transient expression of recombinant proteins’, *Planta*. Springer Verlag, 250(2), pp. 463–473. doi: 10.1007/s00425-019-03180-9.

Miao, C. *et al.* (2019) ‘The grain yield modulator miR156 regulates seed dormancy through the gibberellin pathway in rice’, *Nature Communications*. Nature Publishing Group, 10(1), p. 3822. doi: 10.1038/s41467-019-11830-5.

Ntui, V. O., Tripathi, J. N. and Tripathi, L. (2020) ‘Robust CRISPR/Cas9 mediated genome editing tool for banana and plantain (*Musa spp*.)’, *Current Plant Biology*. Elsevier B.V., 21, p. 100128. doi: 10.1016/j.cpb.2019.100128.

Odipio, J. *et al.* (2017) ‘Efficient CRISPR/cas9 genome editing of phytoene desaturase in cassava’, *Frontiers in Plant Science*. Frontiers Media S.A., 8. doi: 10.3389/fpls.2017.01780.

Okada, A. *et al.* (2019) ‘CRISPR/Cas9-mediated knockout of *Ms1* enables the rapid generation of male-sterile hexaploid wheat lines for use in hybrid seed production’, *Plant Biotechnology Journal*. Blackwell Publishing Ltd, 17(10), pp. 1905–1913. doi: 10.1111/pbi.13106.

Oliva, R. *et al.* (2019) ‘Broad-spectrum resistance to bacterial blight in rice using genome editing’, *Nature Biotechnology*. Nature Publishing Group, 37(11), pp. 1344–1350. doi: 10.1038/s41587-019-0267-z.

Ouyang, L., Ma, M. and Li, L. (2020) ‘An efficient transgene-free DNA-editing system for Arabidopsis using a fluorescent marker’, *Biotechnology Letters*. Springer, 42(2), pp. 313–318. doi: 10.1007/s10529-019-02778-z.

Papikian, A. *et al.* (2019) ‘Site-specific manipulation of Arabidopsis loci using CRISPR-Cas9 SunTag systems’, *Nature Communications*. Nature Publishing Group, 10(1). doi: 10.1038/s41467-019-08736-7.

Peng, A. *et al.* (2017) ‘Engineering canker-resistant plants through CRISPR/Cas9-targeted editing of the susceptibility gene CsLOB1 promoter in citrus’, *Plant Biotechnology Journal*. Blackwell Publishing Ltd, 15(12), pp. 1509–1519. doi: 10.1111/pbi.12733.

Pérez, L. *et al.* (2019) ‘CRISPR/Cas9 mutations in the rice Waxy/GBSSI gene induce allele-specific and zygosity-dependent feedback effects on endosperm starch biosynthesis’, *Plant Cell Reports*. Springer Verlag, 38(3), pp. 417–433. doi: 10.1007/s00299-019-02388-z.

Permyakova, N. V. *et al.* (2019) ‘CRISPR/Cas9-mediated gfp gene inactivation in Arabidopsis suspension cells’, *Molecular Biology Reports*. Springer, 46(6), pp. 5735–5743. doi: 10.1007/s11033-019-05007-y.

Pompili, V. *et al.* (2020) ‘Reduced fire blight susceptibility in apple cultivars using a high-efficiency CRISPR/Cas9-FLP/FRT-based gene editing system’, *Plant Biotechnology Journal*. Blackwell Publishing Ltd, 18(3), pp. 845–858. doi: 10.1111/pbi.13253.

Qin, L. *et al.* (2020) ‘High-efficient and precise base editing of C•G to T•A in the allotetraploid cotton (*Gossypium hirsutum*) genome using a modified <scp>CRISPR</scp> /Cas9 system’, *Plant Biotechnology Journal*. Blackwell Publishing Ltd, 18(1), pp. 45–56. doi: 10.1111/pbi.13168.

Qiu, Z. *et al.* (2019) ‘Identification of Candidate HY5-Dependent and -Independent Regulators of Anthocyanin Biosynthesis in Tomato’, *Plant and Cell Physiology*. Oxford University Press, 60(3), pp. 643–656. doi: 10.1093/pcp/pcy236.

Ren, C. *et al.* (2016) ‘CRISPR/Cas9-mediated efficient targeted mutagenesis in Chardonnay (*Vitis vinifera L.)*’, *Scientific Reports*. Nature Publishing Group, 6. doi: 10.1038/srep32289.

Ren, Q. *et al.* (2019) ‘Bidirectional Promoter-Based CRISPR-Cas9 Systems for Plant Genome Editing’, *Frontiers in Plant Science*. Frontiers Media S.A., 10, p. 1173. doi: 10.3389/fpls.2019.01173.

Saika, H. *et al.* (2019) ‘Targeted deletion of rice retrotransposon Tos17 via CRISPR/Cas9’, *Plant Cell Reports*. Springer Verlag, 38(4), pp. 455–458. doi: 10.1007/s00299-018-2357-7.

Shan, Q. *et al.* (2013) ‘Targeted genome modification of crop plants using a CRISPR-Cas system’, *Nature Biotechnology*. Nature Publishing Group, pp. 686–688. doi: 10.1038/nbt.2650.

Shen, L. *et al.* (2017) 'Rapid generation of genetic diversity by multiplex CRISPR/Cas9 genome editing in rice', *Science China Life Sciences*. Science in China Press, 60(5), pp. 506–515. doi: 10.1007/s11427-017-9008-8.

Sun, X. *et al.* (2015) 'Targeted mutagenesis in soybean using the CRISPR-Cas9 system', *Scientific Reports*. Nature Publishing Group, 5. doi: 10.1038/srep10342.

Svitashev, S. *et al.* (2016) 'Genome editing in maize directed by CRISPR-Cas9 ribonucleoprotein complexes', *Nature Communications*. Nature Publishing Group, 7. doi: 10.1038/ncomms13274.

Takeda, Y. *et al.* (2019) 'Lignin characterization of rice *CONIFERALDEHYDE 5-HYDROXYLASE* loss-of-function mutants generated with the CRISPR/Cas9 system', *The Plant Journal*. Blackwell Publishing Ltd, 97(3), pp. 543–554. doi: 10.1111/tpj.14141.

Tang, X. *et al.* (2019) 'Single transcript unit CRISPR 2.0 systems for robust Cas9 and Cas12a mediated plant genome editing', *Plant Biotechnology Journal*. Blackwell Publishing Ltd, 17(7), pp. 1431–1445. doi: 10.1111/pbi.13068.

Tian, S. *et al.* (2017) 'Efficient CRISPR/Cas9-based gene knockout in watermelon', *Plant Cell Reports*. Springer Verlag, 36(3), pp. 399–406. doi: 10.1007/s00299-016-2089-5.

Tripathi, J. N. *et al.* (2019) 'CRISPR/Cas9 editing of endogenous banana streak virus in the B genome of Musa spp. overcomes a major challenge in banana breeding', *Communications Biology*. Nature Research, 2(1). doi: 10.1038/s42003-019-0288-7.

Ueta, R. *et al.* (2017) 'Rapid breeding of parthenocarpic tomato plants using CRISPR/Cas9', *Scientific Reports*. Nature Publishing Group, 7(1). doi: 10.1038/s41598-017-00501-4.

Veillet, F., Chauvin, L., *et al.* (2019) 'The Solanum tuberosum GBSSI gene: a target for assessing gene and base editing in tetraploid potato', *The Solanum tuberosum GBSSI gene: a target for assessing gene and base editing in tetraploid potato*. Cold Spring Harbor Laboratory, p. 628107. doi: 10.1101/628107.

Veillet, F., Perrot, L., *et al.* (2019) 'Transgene-Free Genome Editing in Tomato and Potato Plants Using Agrobacterium-Mediated Delivery of a CRISPR/Cas9 Cytidine Base Editor', *International Journal of Molecular Sciences*. Multidisciplinary Digital Publishing Institute (MDPI), 20(2). doi: 10.3390/IJMS20020402.

Wang, L. *et al.* (2017) 'Reduced drought tolerance by CRISPR/Cas9-mediated SlMAPK3 mutagenesis in tomato plants', *Journal of Agricultural and Food Chemistry*. American Chemical Society, 65(39), pp. 8674–8682. doi: 10.1021/acs.jafc.7b02745.

Wang, L. *et al.* (2019) 'CRISPR/Cas9-mediated editing of CsWRKY22 reduces susceptibility to Xanthomonas citri subsp. citri in Wanjincheng orange (Citrus sinensis (L.) Osbeck)', *Plant Biotechnology Reports*. Springer Tokyo, 13(5), pp. 501–510. doi: 10.1007/s11816-019-00556-x..

Wang, S. *et al.* (2015) 'Efficient targeted mutagenesis in potato by the CRISPR/Cas9 system', *Plant Cell Reports*. Springer Verlag, pp. 1473–1476. doi: 10.1007/s00299-015-1816-7.

Wang, W.-C. *et al.* (2019) 'Response Regulators 9 and 10 Negatively Regulate Salinity Tolerance in Rice'. doi: 10.1093/pcp/pcz149.

Wang, X. *et al.* (2019) 'Disruption of an amino acid transporter LHT1 leads to growth inhibition and low yields in rice', *BMC Plant Biology*. BioMed Central Ltd., 19(1), p. 268. doi: 10.1186/s12870-019-1885-9.

Wang, X. *et al.* (2018) 'CRISPR/Cas9-mediated efficient targeted mutagenesis in grape in the first generation', *Plant Biotechnology Journal*. Blackwell Publishing Ltd, 16(4), pp. 844–855. doi: 10.1111/pbi.12832.

Wang, Z. et al. (2018) 'Optimized paired-sgRNA/Cas9 cloning and expression cassette triggers high-efficiency multiplex genome editing in kiwifruit', Plant Biotechnology Journal. Blackwell Publishing Ltd, 16(8), pp. 1424–1433. doi: 10.1111/pbi.12884.

Woo, J. W. *et al.* (2015) 'DNA-free genome editing in plants with preassembled CRISPR-Cas9 ribonucleoproteins', *Nature Biotechnology*. Nature Publishing Group, 33(11), pp. 1162–1164. doi: 10.1038/nbt.3389.

Wu, Y. *et al.* (2019) 'Increasing Cytosine Base Editing Scope and Efficiency With Engineered Cas9-PmCDA1 Fusions and the Modified sgRNA in Rice', *Frontiers in Genetics*. Frontiers Media S.A., 10(APR), p. 379. doi: 10.3389/fgene.2019.00379.

Xie, K. and Yang, Y. (2013) 'RNA-Guided Genome Editing in Plants Using a CRISPR–Cas System', *Molecular Plant*. Cell Press, 6(6), pp. 1975–1983. doi: 10.1093/MP/SST119.

Xiong, X. *et al.* (2019) 'Efficient genome editing of Brassica campestris based on the CRISPR/Cas9 system', *Molecular Genetics and Genomics*. Springer Verlag, 294(5), pp. 1251–1261. doi: 10.1007/s00438-019-01564-w.

Xu, R. *et al.* (2017) ‘Generation of targeted mutant rice using a CRISPR-Cpf1 system’, *Plant Biotechnology Journal*. Blackwell Publishing Ltd, 15(6), pp. 713–717. doi: 10.1111/pbi.12669.

Xu, Wenjie *et al.* (2019) ‘Comprehensive analysis of CRISPR/Cas9-mediated mutagenesis in arabidopsis thaliana by genome-wide sequencing’, *International Journal of Molecular Sciences*. MDPI AG, 20(17). doi: 10.3390/ijms20174125.

Xu, Wen *et al.* (2019) ‘Multiplex nucleotide editing by high-fidelity Cas9 variants with improved efficiency in rice’, *BMC Plant Biology*. BioMed Central, 19(1). doi: 10.1186/s12870-019-2131-1.

Yang, H. *et al.* (2017) ‘CRISPR/Cas9-mediated genome editing efficiently creates specific mutations at multiple loci using one sgRNA in Brassica napus’, *Scientific Reports*. Nature Publishing Group, 7(1). doi: 10.1038/s41598-017-07871-9.

Yang, Y. *et al.* (2017) ‘The RNA editing factor SLORRM4 is required for normal fruit ripening in tomato1’, *Plant Physiology*. American Society of Plant Biologists, 175(4), pp. 1690–1702. doi: 10.1104/pp.17.01265.

Yin, X. *et al.* (2017) ‘CRISPR-Cas9 and CRISPR-Cpf1 mediated targeting of a stomatal developmental gene EPFL9 in rice’, *Plant Cell Reports*. Springer Verlag, 36(5), pp. 745–757. doi: 10.1007/s00299-017-2118-z.

Young, J. *et al.* (2019) ‘CRISPR-Cas9 Editing in Maize: Systematic Evaluation of Off-target Activity and Its Relevance in Crop Improvement’, *Scientific Reports*. Nature Publishing Group, 9(1), p. 6729. doi: 10.1038/s41598-019-43141-6.

Yu, Q. H. *et al.* (2017) ‘CRISPR/Cas9-induced Targeted Mutagenesis and Gene Replacement to Generate Long-shelf Life Tomato Lines’, *Scientific Reports*. Nature Publishing Group, 7(1). doi: 10.1038/s41598-017-12262-1.

Yu, T. *et al.* (2019) ‘Genome-wide identification of long non-coding RNA targets of the tomato MADS box transcription factor RIN and function analysis’, *Annals of Botany*, 123, pp. 469–482. doi: 10.1093/aob/mcy178.

Zhai, Y. et al. (2019) ‘CRISPR/Cas9-mediated genome editing reveals differences in the contribution of INDEHISCENT homologues to pod shatter resistance in Brassica napus L.’, Theoretical and Applied Genetics. Springer Verlag, 132(7), pp. 2111–2123. doi: 10.1007/s00122-019-03341-0.

Zhang, F. *et al.* (2017) ‘Rapid and efficient CRISPR/Cas9 gene editing in Citrus using the YAO promoter’, *Plant Cell Reports*. Springer Verlag, 36(12), pp. 1883–1887. doi: 10.1007/s00299-017-2202-4.

Zhang, Hui *et al.* (2014) ‘The CRISPR/Cas9 system produces specific and homozygous targeted gene editing in rice in one generation’, *Plant Biotechnology Journal*. John Wiley & Sons, Ltd (10.1111), 12(6), pp. 797–807. doi: 10.1111/pbi.12200.

Zhang, J. *et al.* (2020) ‘A unique chromosome translocation disrupting *ClWIP1* leads to gynoecy in watermelon’, *The Plant Journal*. Blackwell Publishing Ltd, 101(2), pp. 265–277. doi: 10.1111/tpj.14537.

Zhang, K. *et al.* (2019) ‘Effective editing for lysophosphatidic acid acyltransferase 2/5 in allotetraploid rapeseed (*Brassica napus L.*) using CRISPR-Cas9 system.’, *Biotechnology for biofuels*. BioMed Central Ltd., 12(1), p. 225. doi: 10.1186/s13068-019-1567-8.

Zhang, S. *et al.* (2018) ‘Targeted mutagenesis using the Agrobacterium tumefaciens-mediated CRISPR-Cas9 system in common wheat’, *BMC Plant Biology*. BioMed Central Ltd., 18(1), p. 302. doi: 10.1186/s12870-018-1496-x.

Zhang, Y. *et al.* (2016) ‘Efficient and transgene-free genome editing in wheat through transient expression of CRISPR/Cas9 DNA or RNA’, *Nature Communications*. Nature Publishing Group, 7. doi: 10.1038/ncomms12617.

Zhang, Y. *et al.* (2017) ‘Simultaneous modification of three homoeologs of *TaEDR1* by genome editing enhances powdery mildew resistance in wheat’, *The Plant Journal*, 91(4), pp. 714–724. doi: 10.1111/tpj.13599.

Zhang, Z. *et al.* (2019) ‘Development of an *Agrobacterium* -delivered CRISPR/Cas9 system for wheat genome editing’, *Plant Biotechnology Journal*. Blackwell Publishing Ltd, 17(8), pp. 1623–1635. doi: 10.1111/pbi.13088.

Zheng, M. *et al.* (2020) ‘Knockout of two *Bna MAX 1* homologs by CRISPR/Cas9-targeted mutagenesis improves plant architecture and increases yield in rapeseed (*Brassica napus* L.)’, *Plant Biotechnology Journal*. Blackwell Publishing Ltd, 18(3), pp. 644–654. doi: 10.1111/pbi.13228.

Zhong, Z. et al. (2019) ‘Improving Plant Genome Editing with High-Fidelity xCas9 and Non-canonical PAM-Targeting Cas9-NG’. Molecular Plant. 12(7). Pp 1027-1036. doi: 10.1016/j.molp.2019.03.011.

Zhou, J. *et al.* (2017) ‘CRISPR-cas9 based genome editing reveals new insights into microRNA function and regulation in rice’, *Frontiers in Plant Science*. Frontiers Media S.A., 8. doi: 10.3389/fpls.2017.01598.

Zhu, C. *et al.* (2019) ‘Genome sequencing and CRISPR/Cas9 gene editing of an early flowering Mini-Citrus (*Fortunella hindsii*)’, *Plant Biotechnology Journal*. Blackwell Publishing Ltd, 17(11), pp. 2199–2210. doi: 10.1111/pbi.13132.

Zhu, Y. *et al.* (2019) ‘CRISPR/Cas9-mediated functional recovery of the recessive *rc* allele to develop red rice’, *Plant Biotechnology Journal*. Blackwell Publishing Ltd, 17(11), pp. 2096–2105. doi: 10.1111/pbi.13125.

Zong, Y. *et al.* (2017) 'Precise base editing in rice, wheat and maize with a Cas9-cytidine deaminase fusion', *Nature Biotechnology*. Nature Publishing Group, 35(5), pp. 438–440. doi: 10.1038/nbt.3811.
